# Evaluation of disproportionately enlarged subarachnoid-space hydrocephalus in progressive supranuclear palsy

**DOI:** 10.1093/braincomms/fcaf206

**Published:** 2025-06-03

**Authors:** Mu-Hui Fu, Jeffrey L Gunter, Ryota Satoh, Rodolfo G Gatto, Farwa Ali, Heather M Clark, Julie A Stierwalt, Mary M Machulda, Yehkyoung C Stephens, Hossam Youssef, Nha Trang Thu Pham, Clifford R Jack, Val J Lowe, Keith A Josephs, Jennifer L Whitwell

**Affiliations:** Department of Neurology, Mayo Clinic, Rochester, MN 55905, USA; Department of Neurology, Kaohsiung Chang Gung Memorial Hospital and Chang Gung University College of Medicine, Kaohsiung 83301, Taiwan; Department of Radiology, Mayo Clinic, Rochester, MN 55905, USA; Department of Radiology, Mayo Clinic, Rochester, MN 55905, USA; Department of Neurology, Mayo Clinic, Rochester, MN 55905, USA; Department of Neurology, Mayo Clinic, Rochester, MN 55905, USA; Department of Neurology, Mayo Clinic, Rochester, MN 55905, USA; Department of Neurology, Mayo Clinic, Rochester, MN 55905, USA; Department of Psychiatry and Psychology, Mayo Clinic, Rochester, MN 55905, USA; Department of Neurology, Mayo Clinic, Rochester, MN 55905, USA; Department of Neurology, Mayo Clinic, Rochester, MN 55905, USA; Department of Radiology, Mayo Clinic, Rochester, MN 55905, USA; Department of Radiology, Mayo Clinic, Rochester, MN 55905, USA; Department of Radiology, Mayo Clinic, Rochester, MN 55905, USA; Department of Neurology, Mayo Clinic, Rochester, MN 55905, USA; Department of Radiology, Mayo Clinic, Rochester, MN 55905, USA

**Keywords:** normal pressure hydrocephalus, corticobasal syndrome, Evans’ index, periventricular white matter hyperintensities, deep white matter hyperintensities

## Abstract

Normal pressure hydrocephalus is typically defined by the triad of gait disturbance, cognitive impairment, and urinary incontinence, and is characterized on MRI by disproportionately enlarged subarachnoid-space. Gait disturbance is also a commonly reported symptom in Parkinsonian disorders, especially progressive supranuclear palsy, although the frequency, clinical significance and mechanisms of hydrocephalus in these disorders are unclear. We aimed to assess the prevalence of hydrocephalic MRI parameters in a large cohort of Parkinsonian disorders and evaluate associations with clinical features and abnormalities on MRI and PET. Two hundred and thirty-eight participants with a Parkinsonian disorder, including 181 progressive supranuclear palsy, 36 corticobasal syndrome and 21 Parkinson’s disease, were enrolled from Mayo Clinic by the Neurodegenerative Research Group between September 2009 to October 2023. Automated detection of disproportionately enlarged subarachnoid-space hydrocephalus (D) was applied and using Evans’ index (E) cut-off point >0.3, participants were classified based on both measures as imaging-suggestive of hydrocephalus (D+E+), enlarged subarachnoid-space only (D+E−), large Evans’ index only (D−E+) and no imaging evidence of hydrocephalus (D−E−). Demographic, clinical and imaging features, including magnetic resonance parkinsonism index, cortical and subcortical volumes, white matter hyperintensities, diffusion tractography metrics, and metabolism on PET, were compared across groups. Among the 238 participants, 24 had borderline subarachnoid space scores and were excluded. The remaining 214 participants were classified as: D+E+ (*n* = 20, 9%); D+E− (*n* = 8, 4%); D−E+ (*n* = 71, 33%) and D−E− (*n* = 115, 54%). Among the progressive supranuclear palsy participants, 11% were D+E+, 4% D+E−, 34% D−E+ and 51% D−E−. Most cases (*n* = 18) in the imaging-suggestive of hydrocephalus D+E+ group had progressive supranuclear palsy. The D+E+ participants were older, had more disorientation, more downgaze palsy, greater midbrain and cortical atrophy, lower striatal metabolism, greater degeneration of long-range white matter tracts, larger cistern areas and more periventricular and deep white matter hyperintensities compared to the D−E− participants without imaging evidence of hydrocephalus. The D+E− participants had the highest metabolism in the paracentral lobule and superior parietal gyrus. The D−E+ participants showed worse disease severity and greater midbrain and cortical atrophy compared to the D−E− participants. These findings demonstrate that disproportionately enlarged subarachnoid-space hydrocephalus occurs in ∼15% of progressive supranuclear palsy participants, and is associated with worse clinical and imaging outcomes, as well as white matter hyperintensities. We hypothesize that disproportionately enlarged subarachnoid-space may be a mechanistic byproduct of degeneration and subsequent cerebrospinal fluid flow re-distribution in progressive supranuclear palsy.

## Teaser

Fu *et al*. investigate MRI features of normal pressure hydrocephalus (disproportionately enlarged subarachnoid-space and Evans’ index) in Parkinsonian disorders and report that these features were associated with progressive supranuclear palsy, as well as unique clinical and neuroimaging pictures. These findings led to new speculation of progressive supranuclear palsy pathomechanism.

## Graphical Abstract

**Graphical Abstract:**
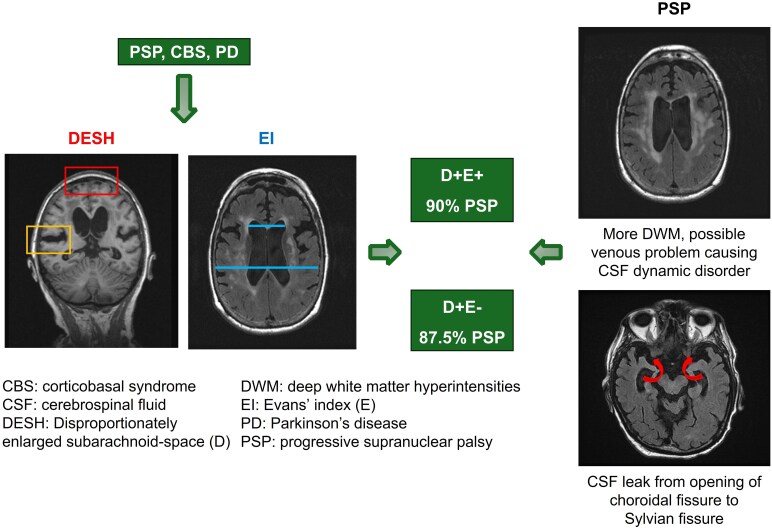

## Introduction

Since first unravelled by Hakim in 1964, normal pressure hydrocephalus (NPH) is characterized by the typical clinical triad of gait disturbance, cognitive impairment and urinary incontinence, with ventriculomegaly as the radiological hallmark.^1^ The prevalence varies widely from 0.01 to 0.20%, especially in the elderly.^2,3^ A prevalence of 1.5% was even reported in those aged 70 years or older.^4^

The mechanism of NPH remains a puzzle, particularly given the presence of multiple comorbid conditions observed in elderly populations. Prior studies showed that NPH is commonly associated with Alzheimer’s disease, cardiovascular disease, stroke and vascular risk factors.^5,6^ Parkinsonian symptoms are frequent in NPH and have long been on the lower body Parkinsonism differential list.^7^ The overlapped symptoms blur the margin between NPH and Parkinson’s disease, dementia with Lewy bodies, vascular parkinsonism and atypical parkinsonism like progressive supranuclear palsy (PSP), corticobasal syndrome (CBS) and multiple system atrophy. Among them, PSP most often clinically and radiologically mimics NPH.^8-10^ Based on the American–European guideline proposed in 2005, gait disturbance is the cardinal feature for the diagnosis of ‘probable’ NPH.^11^ According to the third edition of Japanese NPH guidelines,^12^ one symptom of either gait/urination/cognition with accompanying ventriculomegaly fits the classification of possible NPH. Gait problems with frequent falling are similarly early features of PSP. Based on the experiences of the Queen Square Brain Bank and the University of Cincinnati, three-quarters and two-thirds of their presumed NPH cases were proved to be PSP after autopsy.^13,14^ Several studies tried to discriminate NPH from PSP through clinical and imaging characteristics, but had conflicting and inconsistent results.^15-17^

On diagnosing NPH, an Evans’ index (EI) > 0.3 is the most popular screening tool for the determination of ventriculomegaly. EI is the ratio of the maximal width of the frontal horns to the maximal inner skull diameter at the same level. Other imaging features listed in the American–European guidelines, including sharp callosal angle (CA, the angle between the lateral ventricles on the coronal plane of the posterior commissure), enlargement of temporal horns, and periventricular signal changes.^11^ The debut of an imaging feature known as disproportionately enlarged subarachnoid-space hydrocephalus (DESH) was first published as a diagnostic guideline in the second edition of the Japanese diagnostic guidelines for diagnosing NPH in 2012,^18^ and was retained in the third edition guidelines.^12^ In one memory clinic-based study in Singapore, the prevalence of DESH in the community was 1.1% and increased with age.^19^ It is defined as dilated Sylvian fissures, shrinkage of the subarachnoid space at high convexities (high-convexity tightness), along with ventriculomegaly (EI > 0.3).^20^ In the SINPHONI-2 study, two groups of NPH patients received shunting at different times, revealing that the presence of DESH indicated higher chances of a positive tap test and benefit from shunting.^12^ It has also been proposed that preoperative DESH correlates with good short-term postoperative outcomes after 1 year.^21^ However, its clinical application as a diagnostic or prognostic marker is being challenged due to the low negative predictive value.^22,23^ Furthermore, DESH is an imaging feature observed during the MRI volumetry-based investigation, and the plausible mechanism of its occurrence is limited.^24^ Using 3D MRI, it has been proposed that the DESH is caused by direct cerebrospinal fluid (CSF) communication between the inferior horn of the lateral ventricles and the ambient cistern at the choroidal fissure.^25,26^

Periventricular hyperintensities (PVH) and deep white matter hyperintensities (DWM) are common in NPH patients.^27,28^ Ependymal denudation and elevated interstitial fluid are regarded as the pathophysiological role of PVH,^27^ while demyelination, ischaemic-gliosis, small vessel disease, are presumed causes of DWM.^29^ Studies on white matter tracts with diffusion sequences revealed that the corticospinal tract, corpus callosum, and internal capsule were most affected in NPH when compared with Alzheimer’s disease, Parkinson’s disease or Dementia with Lewy Bodies.^30^

Aside from MRI, new diagnostic tools for NPH emerged recently and raised more intriguing questions. It has been found that on ^123^I-iodoamphetamine brain perfusion SPECT, NPH patients often showed hyperperfusion of the high-convexity area, and termed the finding as convexity apparent hyperperfusion (CAPPAH) sign.^31^ [^18^F]fluorodeoxyglucose (FDG)-PET shows similar regional changes.^32^ With FDG-PET, significant hypometabolism was found in the caudate and putamen with preserved cortical metabolism when comparing NPH with Alzheimer’s disease, Dementia with Lewy Bodies, Parkinson’s disease Dementia and behavioural variant frontotemporal dementia.^33^

The aim of our study was to evaluate DESH and EI in a large cohort of participants with Parkinsonism, including PSP. We were interested in determining the frequency of DESH and EI > 0.3 in this population and whether these NPH signs were associated with other imaging features of NPH, clinical manifestations, or the brain abnormalities related with PSP,^34^ including midbrain and cortical atrophy, white matter degeneration and white matter hyperintensities. Our goal was to alert clinicians about the diagnosis of NPH and its overlap with Parkinsonism, and to provide more guidance while making management decisions based on imaging features. We hypothesize that a substantial proportion of NPH is the masquerade of PSP, and more considerations should be taken into while treating these patients.

## Materials and methods

### Participants

Two hundred and thirty-eight participants with a Parkinsonian disorder, including 181 with PSP, 36 with CBS, and 21 with Parkinson’s disease, were recruited from the Neurodegenerative Research Group (NRG) at Mayo Clinic, Rochester, MN, between September 2009 and October 2023. The clinical diagnoses met the published criteria at the time of enrollment,^35,36^ and were determined at a consensus meeting involving two movement disorders neurologists with expertise in degenerative Parkinsonian disorders (K.A.J. and F.A.). The PSP clinical variants present in the cohort included PSP-Richardson syndrome (PSP-RS) (*n* = 96, 53%), PSP-Parkinsonism (PSP-P) (*n* = 30, 16.6%), PSP-corticobasal syndrome (PSP-CBS) (*n* = 16, 8.8%), PSP-speech/language (PSP-SL) (*n* = 16, 8.8%), PSP-progressive gait freezing (PSP-PGF) (*n* = 10, 5.6%), PSP-frontal (PSP-F) (*n* = 8, 4.4%), PSP-postural instability (PSP-PI) (*n* = 4, 2.2%) and PSP-oculomotor (PSP-OM) (*n* = 1, 0.6%). Fifty-two healthy individuals were also recruited by NRG during the same time period to serve as controls.

### Clinical and neurological evaluations

All participants underwent extensive neurological and neuropsychological evaluations. The tests included Montreal Cognitive Assessment (MoCA)^37^ for general cognition, the Apraxia of Speech Rating Scale (ASRS), the Movement Disorder Society-sponsored revision of the Unified Parkinson’s Disease Rating Scale Part III (UPDRS-III)^38^ for assessment of motor severity, PSP Saccadic Impairment Scale (PSIS)^39^ for ocular motor impairment, PSP Rating Scale^40^ for PSP disease severity, Frontal Assessment Battery (FAB)^41^ for executive function, and test of upper limb apraxia (TULIA)^42^ for evaluation of limb apraxia. The subscores of urine incontinence in Part I History, Mentations of Part II, Ocular Motor of Part IV, and Gait and midline (PSP Rating Scale_GM) of Part VI were extracted from the PSP Rating Scale.

### Neuroimaging

#### Image acquisition

All participants underwent 3 Tesla volumetric MRI on GE or Siemens scanners at Mayo Clinic, Rochester, MN that included a magnetization prepared rapid gradient echo (MP-RAGE) sequence, diffusion tensor imaging (DTI) sequence and a T2-weighted or fluid-attenuated inversion recovery (FLAIR) sequence.^43,44^ MRI acquisition parameters are provided in Supplementary Table 1. All scans underwent quality control assessments. Of the 238 participants, 123 had also undergone FDG-PET. FDG-PET scans were acquired with a PET/CT scanner (GE Healthcare) while operating in 3D mode. Participants were injected with ^18^F-FDG of ∼459 MBq (range 367–576 MBq), and after a 30-min uptake period an 8-min ^18^F-FDG scan was performed.

#### DESH and Evans’ index

Automated measurement of DESH was performed using a previously published and validated machine learning algorithm.^45^ The algorithm was trained and tested using 1597 participants from the Mayo Clinic Study of Aging, an epidemiologically recruited sample of participants from Olmsted County, MN, the majority of which were cognitively normal. None of the Parkinsonian participants in the current study were included in the training or test cohorts. In brief, SPM12 was used to segment the CSF on T1-weighted MPRAGE images, and volumes of 123 sulcal regions-of-interest (relevant to high-convexity tightness) were normalized to total intra-cranial volume. The sulcal regions that best correlated with visually assessed DESH were input into a support vector machine model. Ventriculomegaly and dilated Sylvian fissures were weighed less because these features can sometimes be seen in aging processes. The output of the algorithm gave a ‘DESH pattern score’, which can be considered a DESH probability score. The score had a decision boundary at zero, negative scores if no pattern was found, and increasingly positive scores indicated the presence of more ‘DESH-like’ features. Participants with scores in the range 0 to 1 were a mix of the dominant class (DESH negative) and the minority class (DESH positive). Thus, an abnormal DESH cut-point was set at >1.0.^46^ Cases with scores between 0 and 1 were excluded from the study given the high degree of uncertainty.^45,47^ Examples of various automated DESH pattern scores are shown in Supplementary Fig. 1. Visual assessment of DESH was determined by a neurologist (F.M.H.) blinded to all clinical data.

**Figure 1:**
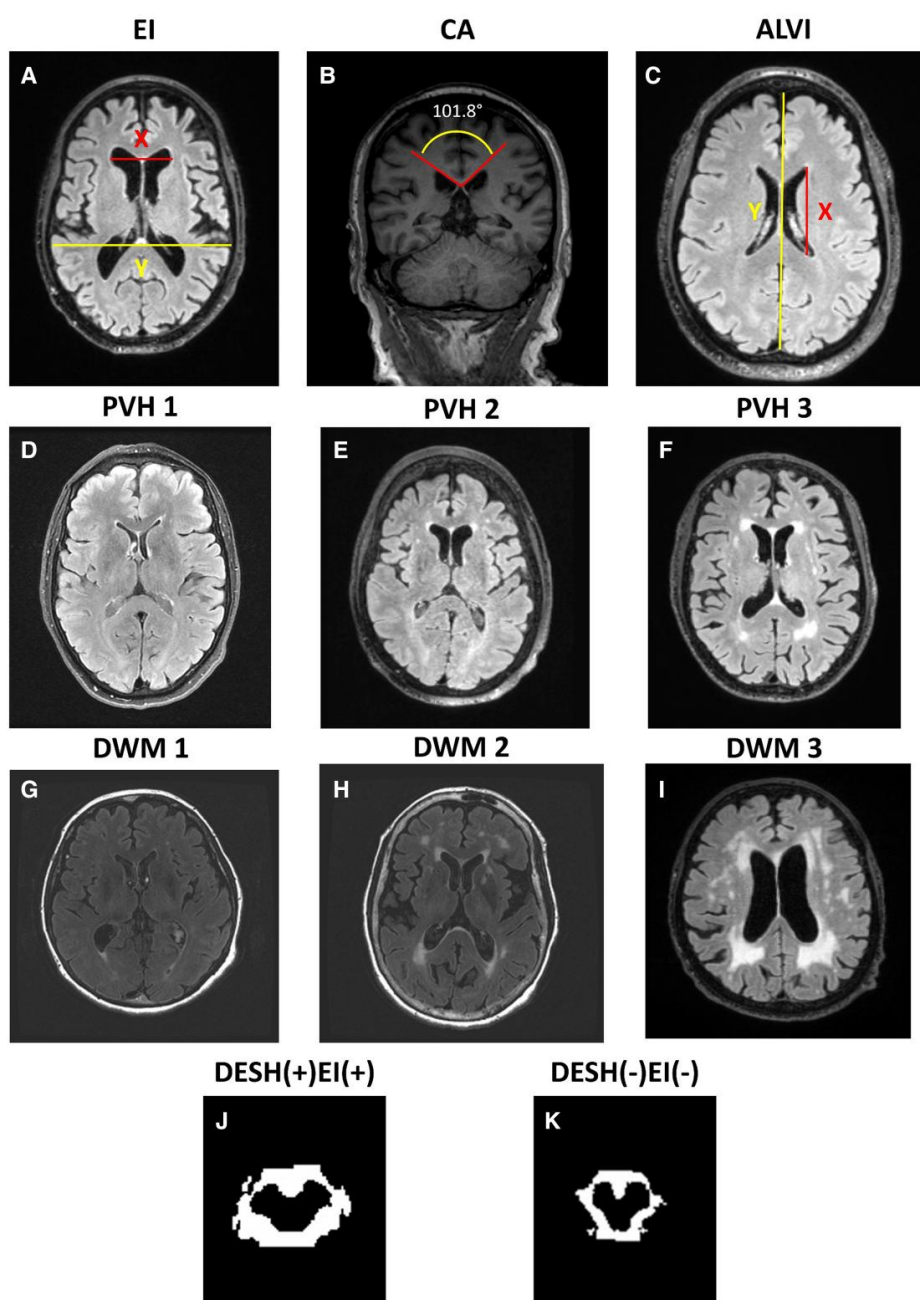
**Examples of manually MRI measurements.** (**A**) Evans’ index (EI): maximum width of the frontal horns of the lateral ventricles (X)/the maximal internal diameter of the skull at the same level (Y) = 0.30. (**B**) Callosal angle (CA): measured on a coronal image perpendicular to the anterior commissure–posterior commissure plane at the level of the posterior commissure. (**C**) Anteroposterior diameter of the lateral ventricle index (ALVI): anteroposterior diameter of lateral ventricle (X)/maximal width of the anteroposterior inner diameter of the skull (Y) = 0.33. (**D–F**) Fazekas scale of PVH 1–3: 1 = ‘caps or pencil-thin-lining’, 2 = ‘smooth halo or thin band’, and 3 = ‘irregular PVH extending into the deep white matter or broad band’. (**G–I**) Fazekas scale of DWM, grade 0 = ‘absent’, 1 = ‘punctate’, 2 = ‘beginning of confluence’, and 3 = ‘confluent’. (**J, K**) Example imaging of manually measured cisterns in D+E+ and D−E− participants. Cistern areas include crural, ambient and quadrigeminal cisterns.

**Figure 2:**
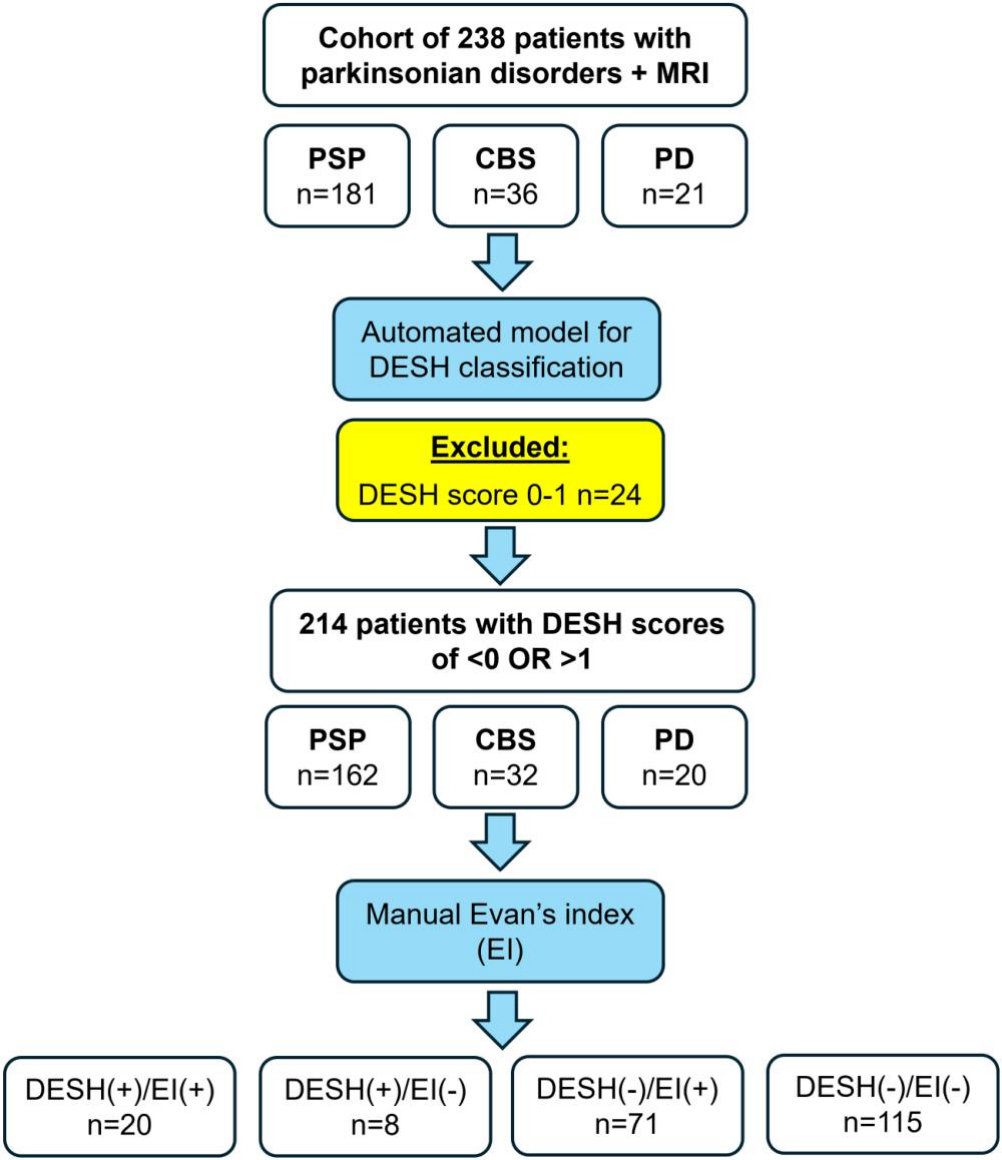
**Flowchart of participants inclusion/exclusion process.** CBS, corticobasal syndrome; DESH, disproportionately enlarged subarachnoid-space hydrocephalus; EI, Evans’ index; PD, Parkinson’s disease; PSP, progressive supranuclear palsy.

Evans’ index was manually measured on the FLAIR scans as the ratio of the maximum width of the frontal horns of the lateral ventricles and the maximal internal diameter of the skull at the same level (Fig. 1A). The intra-rater and inter-rater reliability scores (inter-class correlation coefficients) were 0.98/0.99 for the EI. An EI cut-point of >0.3 was considered abnormal based on the American–European guideline.^11^ The PSP, CBS and Parkinson’s disease participants were classified based on their DESH (D) and EI (E) status as D+E+, D+E−, D−E+ and D−E− (Fig. 2). A flowchart of participants selection and exclusion is depicted in Fig. 2.

#### Manually measured MRI parameters

Several other imaging features were manually measured based on available definitions on MPRAGE, T2 or FLAIR images, including (i) CA: measured on a coronal image perpendicular to the anterior commissure–posterior commissure plane at the level of the posterior commissure. On this coronal plane, the vertex of the angle was placed in the inferior point of the corpus callosum, and sides of the angle were tangential to the lateral ventricles; (ii) Anteroposterior diameter of the lateral ventricle index (ALVI)^48^: measured as the ratio of the anteroposterior diameter lateral ventricle to the maximal width of the anteroposterior inner diameter of the skull (along the cerebral falx) in the same plane; (iii) MR parkinsonism index (MRPI)^49^: measurements of midbrain area, pons area and superior and middle cerebellar peduncle widths were performed according to a previously published method by one trained and blinded rater (NTTP) using ITK-SNAP software^50^; (4) PVH and deep white matter hyperintensities (DWM) were graded according to Fazekas scale.^51^ For PVH, grade 1 = ‘caps or pencil-thin-lining’, 2 = ‘smooth halo or thin band’ and 3 = ‘irregular PVH extending into the deep white matter or broad band’. For DWM, grade 0 = ‘absent’, 1 = ‘punctate’, 2 = ‘beginning of confluence’ and 3 = ‘confluent’. All measurements were performed blinded to clinical data, and example cases are shown in Fig. 1B-I. The intra- and inter-rater reliability scores were 0.97/0.94 for the CA, 0.89/0.80 for the ALVI, 0.97/0.94 for the MRPI, 50 0.86/0.67 for the PVH and 0.77/0.85 for the DWM.

The CSF areas in the crural, ambient and quadrigeminal cisterns (cm^2^) were manually measured on the MPRAGE by using a MATLAB internal function *roipoly*. Serial axial slices were carefully selected from top to bottom of the midbrain, and then the cisterns and midbrain were manually surrounded in each slice. Only CSF areas were extracted and measured from the surrounding regions by multiplying the CSF masks obtained by tissue segmentation performed in SPM12.^52^ Finally, the cistern areas were calculated by averaging the CSF areas over all selected slices. Cistern areas were calculated for a subset of D+E+ and D−E− participants. Examples of measured cistern areas are shown in Fig. 1J and K.

#### Grey matter volumes

To determine whether the DESH/EI measures are related to cortical atrophy, regional grey matter volumes (GMV) were calculated for all cases. The Mayo Clinic Adult Lifespan Template (MCALT v1.4) atlas was propagated into MPRAGE-space and used to calculate volumes of 47 regions that covered cortical and subcortical structures (Supplementary Table 2). Total intracranial volume (TIV) was used to correct regional GMV for differences in head size.

#### Diffusion tensor imaging

DTI analyses were performed on a subset of 11 D+E+ participants who were matched by age and sex to eleven D−E− participants. One D+E+ GE participant was excluded because there was no age match in the D−E− group. All selected participants had undergone MRI on the GE scanner. The images were initially acquired using a 1.36 mm in-plane resolution and a slice thickness of 2.7 mm. The DWI images were then resampled to a 2 mm isotropic voxel-size by the automatic fibre tracking algorithm implemented in DSI (https://dsi-studio.labsolver.org/doc/gui_t2.html). Diffusion MRI (dMRI) protocol included a spin-echo single shot Echo Planar Imaging sequence. dMRI scans applied two diffusion-weighted values: *b* = 0 and *b* = 1000 s/mm^2^. Forty-one diffusion gradients were included on each shell. DWI motion artefacts were visually inspected for quality, and susceptibility and current distortion artefacts were adjusted using FSL’s top up and eddy algorithms embedded in DSI software and described elsewhere.^53,54^ To ensure its accuracy and avoid outliers, the dataset was inspected by an automatic quality control routine.^55^ In addition, incorporated algorithms were applied to check the accuracy and reliability of the diffusion data.^56^

Datasets from each group were normalized and non-linearly co-registered to a probabilistic tractography atlas via an iterative group-wise registration algorithm and spatially normalized into a MCALT space.^57^ Deterministic random step-size-algorithms were used to propagate and originate the tractography reconstructions.^58^ By means of an ROI-based approach (HCP842 tractography atlas), five million seed points were selected for tracking. Regions where the mean FA was below 0.2 were not considered. Minimal and maximal tract lengths were set to 10 to 300 mm, respectively. Sixteen topology-informed pruning iterations and 16 mm auto-tract tolerance were used for each reconstruction to help remove singular and false positive fibres.^56^ Based on the significant affected grey matter regions and the tracts involved in the pathology as previously described,^59,60^ seven left and right WM tracts were selected for analysis: 1-arcuate fasciculus (AF), 2-extreme capsule (EC), 3-superior longitudinal fasciculus (SLF), 4-inferior frontal-occipital fasciculus (IFOF), 5-inferior longitudinal fasciculus (ILF), 6-uncinate fasciculus (UF) and 7-corticospinal tract (CST). For each tract, we obtained the overall fractional anisotropy (FA) and mean diffusivity (MD) parameters. In instances of gross anatomical degeneration/deformations, manual correction and realignment of GM and WM regions-of-interest were realigned by experienced users familiar with neuroanatomical landmarks as described before.^61^

#### FDG-PET

The MCALT atlas was used to output regional metabolism from the FDG-PET scans. Each FDG-PET scan was co-registered to the participant’s MPRAGE scan using 6 degrees-of-freedom registration in SPM12, and the MPRAGE-space MCALT atlas was used to output region-level data. All values were normalized to the pons to generate standardized uptake value ratios (SUVRs). Based on prior reports of SPECT and FDG-PET characteristics in DESH or NPH,^31,33^ SUVRs of the paracentral lobule, superior parietal gyrus, precuneus, caudate and putamen were extracted and compared across groups. To minimize the contribution of neighbouring regions, SUVRs data were processed with partial volume correction.

### Statistical analysis

Statistical analyses were performed utilizing GraphPad Prism 10.0 (GraphPad Software, Inc., San Diego, CA) or R. Categorical variables were compared across groups using χ^2^ tests (Fishers exact test for cells with small numbers) and continuous variables were compared using nonparametric, Kruskal–Wallis tests. Pair-wise comparisons were corrected for multiple comparisons using Dunn’s test after Kruskal–Wallis tests. In the analysis of GMV, Steel–Dwass test was performed to conduct pair-wise comparisons for those regions-of-interest (ROIs). All the continuous imaging variables were adjusted for age and sex using linear regression. False Discovery Rate (FDR) correction using the Benjamini–Hochberg method was applied to control for multiple comparisons in all analysis. Mann–Whitney U-test was used to analyse FA and MD in DTI. Significance was set at *P* < 0.05 in all analyses. Given that PSP participants made up most of our cohort, all group analyses were also repeated only in the participants with PSP, excluding the participants with CBS and Parkinson’s disease.

## Results

### Study demographics of different Parkinsonism groups

The demographic and clinical features of the PSP, CBS, Parkinson’s disease and control participants are shown in Supplementary Table 3. No differences were observed across the groups in age or sex, or MRI scanner (Supplementary Table 4). On clinical testing, both general cognitive (MoCA) and motor (UPDRS-III) functions were worse in PSP, CBS and Parkinson’s disease participants when compared to controls (*P* < 0.001), and UPDRS-III remained the worst in PSP when comparing across diseased participants. PSP and CBS participants performed worse on the PSP Rating Scale than Parkinson’s disease and controls (*P* < 0.001). The PSIS was, as expected, worse in PSP than CBS, Parkinson’s disease and controls; TULIA was worse in CBS than PSP, Parkinson’s disease and controls.

The proportion of participants with EI > 0.3 was greater in PSP (45.8%) and CBS (36.1%) than controls (*P* < 0.001), although no differences were observed between PSP, CBS and Parkinson’s disease. CA < 90° was observed in seven PSP participants (3.9%) and one CBS participant (2.8%), but not in Parkinson’s disease participants or controls. The distribution of DESH positivity among groups was as follows: PSP 25/181 (13.8%), CBS 0/36 (0%), Parkinson’s disease 3/21 (14.3%) and control 3/52 (5.8%). Visually assessed DESH positivity was PSP 8/181 (4.4%), CBS 0/36 (0%), Parkinson’s disease 1/21 (4.8%) and control 0/52 (0%). Of the seven PSP participants with CA < 90°, five were classified as DESH positive (Supplementary Table 3).

### Clinical demographics of DESH/EI groups

After applying the automated model for DESH classification,^45^ excluding cases with a DESH score between 0 and 1, and combining it with the manually measured EI data, 214 participants were classified into the four DESH/EI groups (Fig. 2). Of the 214 participants, 20 (9%) were D+E+, eight (4%) D+E−, 71 (33%) D−E+, and 115 (54%) D−E−. Age at encounter differed across the four groups, with the D+E+ group older than the D−E− group (*P* = 0.03; Table 1). Sex also differed across groups, with a lower proportion of females in the D−E+ group when compared to the D+E− and D−E− groups (Table 1). There was no difference in disease duration across groups. The D−E+ group performed worse on the MoCA, UPDRS-III, FAB and PSP Rating Scale than the D−E− group, with no other pair-wise differences observed in these measures. No difference was observed in ASRS. In the PSP Rating Scale subscores, there was no difference in the gait/midline subscale and urinary incontinence, but more downgaze problems and disorientation were observed in the D+E+ group compared to the D−E− group (Table 1). Applying the American–European diagnostic guideline,^11^ the proportion of participants meeting possible iNPH criteria was 100% in D+E+ group and 97% in D−E+ group and meeting probable iNPH was 80% in D+E+ group and 79% in D−E+ group (Table 1). When the analysis was limited to PSP participants, the group differences in gender and PSP Rating Scale subscores remained (Supplementary Table 5).

**Table 1:** Demographic data of the four DESH/EI (D/E) groups for the 214 PSP, CBS and Parkinson’s disease participants

Variable	D+E + (*N* = 20)	D+E− (*N* = 8)	D−E+ (*N* = 71)	D−E− (*N* = 115)	FDR corrected *P*-values
Female, *n* (%)	7 (35%)	6 (75%)	21 (29.6%)	61 (53%)	**0.01** ^a,b^
Age at encounter, year	72.9 (69.7, 77.2)	69.8 (63.6, 77.4)	70.4 (66.7, 75.8)	68.6 (63.5, 72.7)	**0**.**03**^c^
Age at onset, year	68 (64.5, 72)	67 (54.5, 72)	67 (63, 71)	64 (59, 69.8)	0.055
Disease duration, year	3.6 (2.1, 6.1)	4.5 (2.5, 6.7)	3.6 (2.3, 4.7)	3.1 (2, 4.9)	0.50
MoCA (30)	23 (21, 26)	23 (18, 26)	22 (19, 24)	24 (22, 26)	**<0**.**01**^b^
UPDRS-III (132)	43 (31, 54)	39 (35, 58)	40 (31, 58)	34 (23, 45)	**<0**.**01**^b^
PSIS (5)	2 (1, 3)	3 (3, 4)	2 (2, 3)	2 (1, 3)	**0**.**04**
FAB (18)	13.5 (12, 15.25)	15.5 (12.5, 17)	13 (11, 15)	15 (13, 16)	**<0**.**01**^b^
ASRS (52)	3.5 (1, 6.25)	4 (2.75, 6.25)	4 (2, 8)	4 (2, 6)	0.62
PSP rating scale (100)	32 (25, 41)	37 (32, 48)	38 (29, 50)	31 (19, 40)	**<0**.**01**^b^
PSP rating scale_GM (20)	13 (8, 15)	10 (9, 14)	12 (6, 15)	9 (4, 14)	0.19
*PSP Rating Scale downgaze score*	1 (1, 3.25)	3 (3, 4)	2 (1, 4)	2 (0, 4)	**<0.05** ^b,c,d,e^
PSP Rating Scale downgaze positive (score ≥ 1), *n*/total (%)	19/20 (95%)	6/7 (85.7%)	57/66 (86.4%)	74/104 (71.2%)	
*PSP Rating Scale disorientation score*	0 (0, 1)	0 (0, 0)	0 (0, 1)	0 (0, 0)	**<0.05** ^b,c^
PSP Rating Scale disorientation positive (score ≥ 1), *n*/total (%)	9/20 (45%)	1/7 (14.3%)	21/66 (31.8%)	13/104 (12.5%)	
*PSP Rating Scale urinary incontinence score*	0 (0, 3)	3 (1, 3.5)	1 (0, 3)	0 (0, 2.25)	0.47
PSP Rating Scale urine incontinence positive (score ≥ 1), *n*/total (%)	9/20 (45%)	5/7 (71.4%)	36/66 (54.5%)	41/104 (39.4%)	
NPH diagnostic criteria					
Possible iNPH criteria, *n* (%)	20 (100%)	0	69 (97%)	0	
Probable iNPH criteria, *n* (%)	16 (80%)	0	56 (79%)	0	

Data shown as *n* (%) or median (range). Categorical variables were compared across groups using χ^2^ tests (Fishers exact test for cells with small numbers) and continuous variables were compared using Kruskal–Wallis tests. Pair-wise comparisons were corrected for multiple comparisons using Dunn’s test. FDR correction was applied to control for multiple comparisons. American–European guideline was used for iNPH diagnostic criteria, in which EI > 0.3 was the pre-requisite of possible and probable iNPH.^11^ Bold values indicate *P* < 0.05.

ASRS, apraxia of speech rating scale; FAB, frontal assessment battery; MoCA, Montreal cognitive assessment; PSIS, PSP saccadic impairment scale; PSP Rating Scale_GM, PSP Rating Scale gait/midline subscore; UPDRS-III, unified Parkinson’s disease rating scale Part III.

Superscript alphabet(s) in *P*-value represents: ^a^DESH(+)EI(−) versus DESH(−)EI(+); ^b^DESH(−)EI(+) versus DESH(−)EI(−); ^c^DESH(+)EI(+) versus DESH(−)EI(−); ^d^DESH(+)EI(+) versus DESH(+)EI(−); ^e^DESH(+)EI(+) versus DESH(−)EI(+); ^f^DESH(+)EI(−) versus DESH(−)EI(−).

The breakdown of clinical diagnoses of each DESH/EI group is shown in Table 2. The D+E+ group consisted of 18 (90%) PSP participants and 2 (10%) Parkinson’s disease participants. Of the 18 PSP participants, the most common clinical variant was PSP-RS, occurring in **45%** of the group, followed by PSP-P in 20%, PSP-CBS in 10%, and then PSP-F, PSP-PGF and PSP-SL in 5% each. When comparing within each clinical variant of PSP, over 10% of the PSP-RS, PSP-CBS, PSP-F, PSP-PGF and PSP-P groups were D+E+, compared to only 7% of PSP-SL. No PSP-PI, PSP-OM or CBS participants were in the D+E+ group (Table 2; Supplementary Fig. 2).

**Table 2:** Parkinsonism subtypes of the four DESH/EI (D/E) groups

Subtype		D+E + (*N* = 20)	D+E− (*N* = 8)	D−E + (*N* = 71)	D−E− (*N* = 115)	Total (214)
PSP	PSP-RS	9 (45%)	2 (25%)	30 (42.3%)	46 (40%)	87
	PSP-CBS	2 (10%)	1 (12.5%)	5 (7%)	5 (4.3%)	13
	PSP-F	1 (5%)	1 (12.5%)	1 (1.4%)	4 (3.5%)	7
	PSP-PGF	1 (5%)	0	0	8 (7%)	9
	PSP-P	4 (20%)	3 (37.5%)	10 (14.1%)	10 (8.7%)	27
	PSP-SL	1 (5%)	0	9 (12.7%)	5 (4.3%)	15
	PSP-PI	0	0	0	3 (2.6%)	3
	PSP-OM	0	0	0	1 (0.9%)	1
Total PSP	*N*	18	7	55	82	162
	% within DESH/EI	18/20 = 90%	7/8 = 87.5%	55/71 = 77.5%	82/115 = 71.3%	
	% of total PSP	18/162 = 11%	7/162 = 4%	55/162 = 34%	82/162 = 51%	
CBS		0	0	11 (15.5%)	21 (18.3%)	
PD		2 (10%)	1 (12.5%)	5 (7%)	12 (10.4%)	

Data shown as *n* (%).

CBS, corticobasal syndrome; PD, Parkinson’s disease; PSP, progressive supranuclear palsy; PSP-CBS, PSP-corticobasal syndrome; PSP-F, PSP-frontal; PSP-OM, and PSP-oculomotor; PSP-P, PSP-parkinsonism; PSP-PGF, PSP-progressive gait freezing; PSP-PI, PSP-postural instability; PSP-RS, PSP-Richardson syndrome; PSP-SL, PSP-speech/language.

### Imaging features of DESH/EI groups

#### Manually measured parameters

The D+E+ and D−E+ groups both had smaller CA, greater MRPI, and ALVI compared to the D−E− group (Table 3). The combination of crural, ambient and quadrigeminal cistern areas were also larger in the D+E+ group when compared to the D−E− group (*P* < 0.001). Higher frequency of severe PVH and DWM were observed in the D+E+ and D+E− group. The average PVH and DVM scores were greatest in the D+ groups. These results remained the same when the cohort was limited to only PSP participants, except the significance for DWM scores became a trend (Supplementary Table 6).

**Table 3:** Imaging features of the four DESH/EI (D/E) groups

Parameter	D+E + (*N* = 20)	D+E− (*N* = 8)	D−E+ (*N* = 71)	D−E− (*N* = 115)	FDR corrected *P*-values
CA	101.9 (91.9, 114.8)	117.8 (104.5, 124.1)	115.4 (108.3, 122.5)	121 (113.5, 125.7)	**<0.001** ^a,b,c,d^
MRPI	20.45 (15.54, 23.48)	22.10 (14.87, 28.98)	17.20 (12.50, 23.93)	12.85 (9.62, 16.75)	**<0.001** ^c,d,e^
ALVI	0.49 (0.45, 0.56)	0.45 (0.42, 0.47)	0.46 (0.43, 0.50)	0.45 (0.42, 0.47)	**<0.01** ^a,b,c,d^
Cistern areas (cm^2^)	7.71 (7.19, 8.52)	N/A	N/A	6.03 (5.64, 6.46)	**<0.001** ^c^
Fazekas score					
PVH 1	4 (20%)	2 (25%)	21 (29.6%)	60 (52.2%)	
PVH 2	10 (50%)	3 (37.5%)	38 (53.5%)	44 (38.3%)	**0.004** ^c,d,e^
PVH 3	6 (30%)	3 (37.5%)	12 (16.9%)	11 (9.5%)	
Average PVH score	2.10	2.13	1.87	1.57	
DWM 0	1 (5%)	1 (12.5%)	15 (21.1%)	29 (25.2%)	
DWM 1	10 (50%)	3 (37.5%)	35 (49.3%)	63 (54.8%)	
DWM 2	5 (25%)	2 (25%)	20 (28.2%)	20 (17.4%)	**0.002** ^b,c,e,f^
DWM 3	4 (20%)	2 (25%)	1 (1.4%)	3 (2.6%)	
Average DWM score	1.60	1.63	1.10	0.97	

Data shown as *n* (%) or median (range). Group comparisons of CA, MRPI and ALVI were performed using linear regression adjusting for age and gender, and FDR correction was applied to control for multiple comparisons. Categorical variables were compared across groups using chi-square tests (Fishers exact test for cells with small numbers) with FDR correction for multiple comparisons.

Cistern includes crural, ambient, and quadrigeminal cisterns. N/A is not assessed. Bold values indicate *P* < 0.05.

ALVI, anteroposterior diameter of the lateral ventricle index; CA, callosal angle; DWM, deep white matter hyperintensities; MRPI, MR parkinsonism index; PVH, periventricular hyperintensities.

Superscript alphabet(s) in *P*-value represents: ^a^DESH(+)EI(+) versus DESH(+)EI(−); ^b^DESH(+)EI(+) versus DESH(−)EI(+); ^c^DESH(+)EI(+) versus DESH(−)EI(−); ^d^DESH(−)EI(+) versus DESH(−)EI(−); ^e^DESH(+)EI(−) versus DESH(−)EI(−); ^f^DESH(+)EI(−) versus DESH(−)EI(+).

#### Grey matter volumes

Of the 47 regions combining left and right (Supplementary Table 2), 10 showed significant differences across the DESH/EI groups after correction for multiple comparisons (Table 4). The D+E+ group showed smaller GMVs in the orbitofrontal cortex and temporal pole compared to the D−E− group. Conversely, the D+E+ group showed greater GMV in the superior parietal lobe compared to the D+E− and D−E+ groups. The D−E+ group showed smaller GMV in the orbitofrontal cortex, temporal pole, superior parietal lobe and thalamus compared to the D−E− group. Differences across the DESH/EI groups in all 10 regions remained when the cohort was limited to only PSP participants, although fewer pair-wise differences were observed (Supplementary Table 7).

**Table 4:** MRI region-of-interest volumes of the four DESH/EI (D/E) groups

ROI	D+E + (*N* = 20)	D+E− (*N* = 8)	D−E+ (*N* = 70)	D−E− (*N* = 114)	*P*-value adjusted for age and gender
Frontal lobe					
Medial orbitofrontal cortex	0.38 (0.33, 0.41)	0.37 (0.34, 0.38)	0.38 (0.35, 0.42)	0.43 (0.38, 0.46)	**<0.001** ^a,b,c^
Middle frontal gyrus orbital part	0.43 (0.39, 0.46)	0.45 (0.42, 0.47)	0.44 (0.42, 0.49)	0.48 (0.43, 0.51)	**0**.**002**
Inferior frontal gyrus, triangular part	0.46 (0.43, 0.52)	0.46 (0.44, 0.54)	0.49 (0.44, 0.54)	0.53 (0.47, 0.58)	**<0**.**001**
Inferior frontal gyrus, opercular part	0.38 (0.35, 0.44)	0.41 (0.39, 0.43)	0.39 (0.34, 0.44)	0.43 (0.38, 0.47)	**0**.**003**
Temporal lobe					
Temporal pole, middle	0.39 (0.38, 0.43)	0.41 (0.36, 0.44)	0.42 (0.39, 0.47)	0.46 (0.41, 0.51)	**<0.001** ^a,c^
Superior temporal gyrus	1.21 (1.08, 1.27)	1.23 (1.12, 1.35)	1.21 (1.10, 1.32)	1.28 (1.18, 1.41)	**0**.**001**
Parietal lobe					
Superior parietal gyrus	0.83 (0.73, 0.91)	0.71 (0.67, 0.76)	0.74 (0.67, 0.82)	0.79 (0.71, 0.89)	**0.001** ^c,d,e^
Inferior parietal gyrus	0.41 (0.37, 0.49)	0.43 (0.36, 0.47)	0.38 (0.35, 0.44)	0.43 (0.38, 0.48)	**<0**.**001**^c^
Subcortical					
Thalamus	0.31 (0.29, 0.35)	0.38 (0.34, 0.39)	0.34 (0.31, 0.36)	0.37 (0.34, 0.39)	**<0**.**001**^c^
Insula					
Insula	0.83 (0.78, 0.90)	0.87 (0.81, 0.97)	0.85 (0.81, 0.90)	0.89 (0.84, 0.95)	**<0**.**001**

Data shown as median (range). Group comparisons were performed using linear regression adjusting for age and gender, and FDR correction was applied to control for multiple comparisons. ROI volumes are expressed as percentage of TIV. Bold values indicate *P* < 0.05.

Superscript alphabet(s) in *P*-value represents: ^a^DESH(+)EI(+) versus DESH(−)EI(−); ^b^DESH(+)EI(−) versus DESH(−)EI(−); ^c^DESH(−)EI(+) versus DESH(−)EI(−); ^d^DESH(+)EI(+) versus DESH(+)EI(−); ^e^DESH(+)EI(+) versus DESH(−)EI(+); ^f^DESH(+)EI(−) versus DESH(−)EI(+).

#### Diffusion tensor imaging

**Figure 3:**
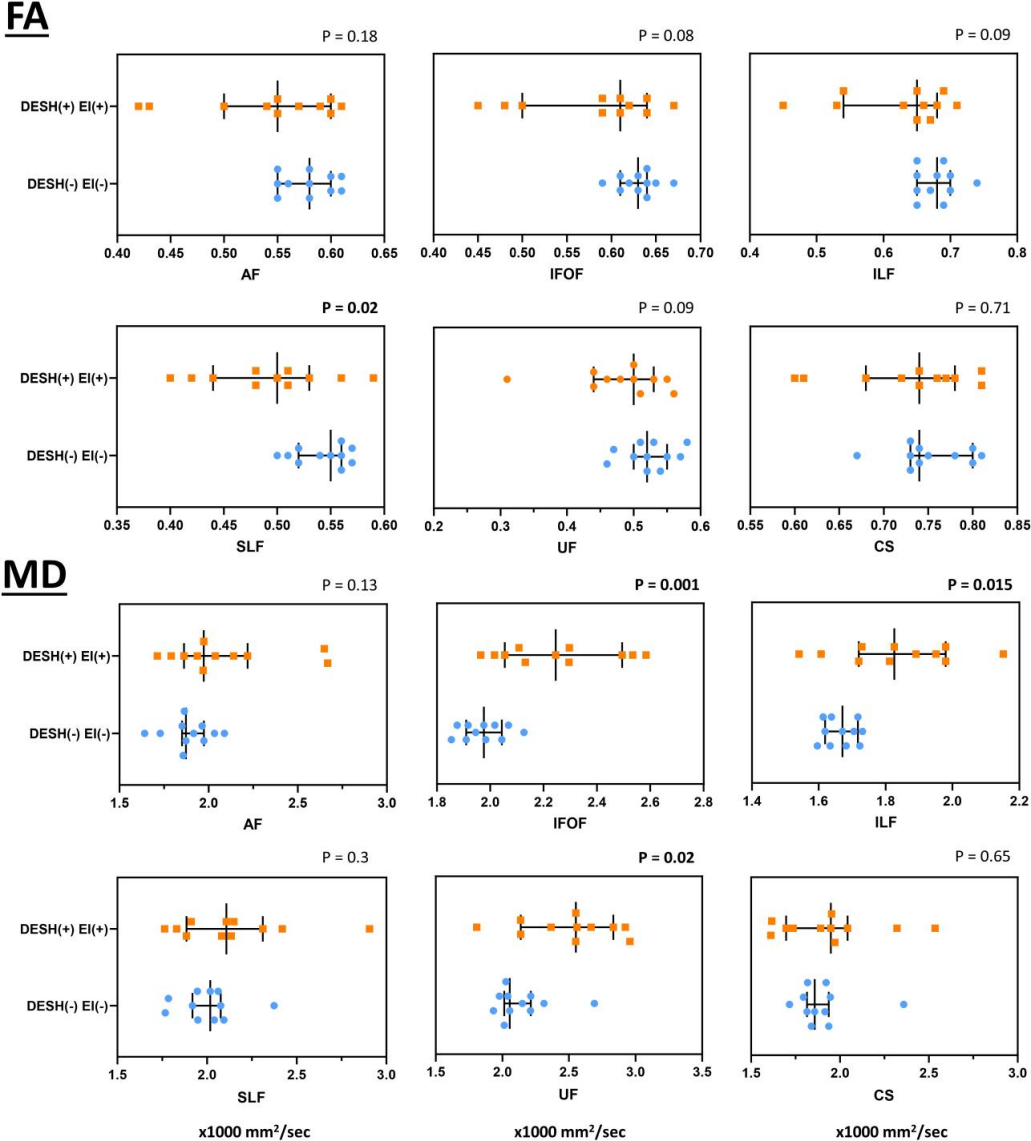
**Results of white matter tracts in D+E+ and D−E− participants.** These plots show fractional anisotropy (FA) and mean diffusivity (MD) metrics for D+E+ and D−E− group, and present median with inter-quartile range. *P*-value is presented at right upper corner of each tract, and marked as bold if reaching significance. AF, arcuate fasciculus; CS, corticospinal tract; IFOF, inferior fronto-occipital fasciculus; ILF, inferior longitudinal fasciculus; UF, uncinate fasciculus. Each group contains eleven participants and were compared using Mann–Whitney U-test.

Compared to the D−E− group, the most striking region of reduced FA in the D+E+ group was observed in the SLF, with trends for differences in the IFOF, ILF and UF. MD was greater in the IFOF, ILF and UF in the D+E+ group compared to D−E− group (Fig. 3). In the analysis of only PSP participants, greater MD in the IFOF in the D+E+ group remained (Supplementary Fig. 3).

#### FDG-PET analysis

FDG-PET was available for 10 D+E+ participants, 5 D+E− participants, 41 D−E + participants, and 67 D−E− participants. The D+E− group showed hypermetabolism in the paracentral lobule and superior parietal gyrus compared to the D−E + and D−E− groups. The D+E+ group showed hypometabolism in the caudate compared to the D+E−, D−E+ and D−E− groups. The D−E + group also showed hypometabolism in the caudate compared to the D−E− group (Table 5). These results remained similar when the cohort was limited to only PSP participants (Supplementary Table 8).

**Table 5:** FDG SUVR of the four DESH/EI (D/E) groups

ROI	D+E + (*N* = 10)	D+E− (*N* = 5)	D−E+ (*N* = 41)	D−E− (*N* = 67)	*P*-value adjusted for age and gender
Paracentral lobule	3.17 (3.02, 3.40)	3.44 (3.30, 3.55)	3.04 (2.90, 3.28)	3.07 (2.93, 3.26)	**0.01** ^a,b^
Superior parietal gyrus	3.25 (3.19, 3.34)	3.56 (3.46, 3.80)	3.17 (3.03, 3.39)	3.29 (3.03, 3.49)	**0.03** ^a,b^
Precuneus	3.31 (3.19, 3.43)	3.68 (3.54, 3.77)	3.33 (3.18, 3.54)	3.48 (3.24, 3.64)	0.41
Caudate	2.50 (2.41, 2.58)	2.82 (2.81, 2.91)	2.65 (2.50, 2.82)	2.81 (2.73, 2.97)	**<0.001^c,d,e,f^**
Putamen	2.77 (2.58, 2.87)	3.11 (2.95, 3.21)	2.91 (2.79, 2.99)	2.93 (2.82, 3.07)	0.07

Data shown as median (range). Group comparisons were performed using linear regression adjusting for age and gender, and False Discovery Rate (FDR) correction was applied to control for multiple comparisons. Bold values indicate *P* < 0.05.

Superscript alphabet(s) in *P*-value represents: ^a^DESH(+)EI(−) versus DESH(−)EI(+); ^b^DESH(+)EI(−) versus DESH(−)EI(−); ^c^DESH(+)EI(+) versus DESH(+)EI(−); ^d^DESH(+)EI(+) versus DESH(−)EI(+); ^e^DESH(+)EI(+) versus DESH(−)EI(−); ^f^DESH(−)EI(+) versus DESH(−)EI(−).

## Discussion

This study evaluated in-depth hydrocephalic imaging parameters among different Parkinsonian disorders, expanded the overlapping imaging features in NPH and PSP, and explored the clinical and neuroimaging associations of DESH in PSP. A high prevalence of NPH imaging features were observed in PSP participants, including DESH. The D+E+ participants have greater midbrain atrophy, larger cistern areas, and more white matter hyperintensities compared to D−E− participants, but D+E− participants had the highest metabolism in the paracentral lobule. The distribution of white matter hyperintensities and proximity of the superior sagittal sinus (SSS) with the paracentral lobule raises the suspicion of venous system involvement in the formation of DESH.

Accumulating evidence demonstrates an intermingling between NPH and PSP. Based on autopsy results from the Queen Square Brain Bank and the University of Cincinnati, three out of four presumed NPH cases were proven to be PSP.^13,14^ One of the classic imaging features of PSP- hummingbird sign,^62^ has also been reported as a supportive diagnosis of NPH.^63^ In fact, a clinical phenotype of PSP with hydrocephalus has been proposed.^8^ Traditionally, NPH and PSP are classified as two distinct diseases. They should be thoroughly distinguished, and several MRI indices have been proposed to differentiate one from another, such as the MRPI, Magnetic Resonance Hydrocephalic Index, inter-peduncular angle, midbrain area and cortical thickness, but all failed.^8,64-66^ In our study, 45.8% of PSP participants have ventriculomegaly (EI > 0.3), and 90% of D+E+ participants in this cohort are PSP. DESH is an imaging feature of NPH with 0.65% in one study of general population in Japan,^67^ 1% among a Singaporean community-based cohort,^19^ and 7% in the Mayo Clinic Study of Aging with the assistance of automated measurement of DESH.^47^ However, the prevalence of DESH in our PSP cohort (13.8%) is 2-fold that of the general elderly population, suggesting there is some meaningful overlap between the two disease entities. However, more studies are needed to determine whether they exist as a comorbidity or in fact the same disease.

The chronic imbalance of CSF production and absorption is believed to be the cause of NPH. In the classical theory of CSF production, CSF is formed predominantly by the choroid plexus in the ventricles, yet this theory is challenged by accumulating evidence suggesting that the formation of CSF, in fact, takes place throughout the entire brain.^68,69^ Regarding the absorption system, the conventional unidirectional CSF flow is from lateral ventricles to the third ventricle to the aqueduct, to the fourth ventricle, through the foramen of Lushka and Magendie into cisterns and the subarachnoid space, then back to SSS, where CSF is absorbed through the arachnoid granulations. Though this pathway is also contradicted by several human and animal studies,^70^ the arachnoid granulations in the SSS are generally accepted as the major site of CSF absorption. SSS is the largest dural venous sinus, receiving blood from several pre-adjoining enlarged venous spaces called lacunae. The largest and most constant lacunae is located in the parietal and posterior frontal regions.^71^ The discovery of the glymphatic system in the paravascular space provides compelling evidence for another route of CSF absorption,^72,73^ and NPH is among the most frequently reported diseases with glymphatic system dysfunction.^74,75^ The glymphatic system is functionally connected to the meningeal lymphatic system, a group of lymphatic vessels running along SSS and exit with the jugular veins through the vascular networks of the skull base.^76,77^ Based on these findings, the intimate relationship between venous drainage and CSF absorption is conspicuous.

Though frequently overlooked when compared with the arterial system, several reports implied the involvement of venous abnormalities as the pathophysiology of NPH.^78,79^ Cerebral venous thrombosis often has the complication of communicating hydrocephalus, especially in patients with deep cerebral venous thrombosis.^80-82^ Altered deep venous drainage in the periventricular area was observed in both normal aging and NPH patients.^83^ The results of brain SPECT and FDG-PET augment this assumption. In SPECT studies, it was found that NPH patients often showed hyperperfusion of the high-convexity area, and the CAPPAH sign was named accordingly.^31^ High-convexity area is a region near the SSS with prominent lacunae. A similar finding was also demonstrated on FDG-PET.^32^ However, the CAPPAH sign was subsequently reported not to reflect true hyperperfusion of regional cerebral blood flow.^31^ Venous congestion is one of the possibilities for the observed hypermetabolism on FDG-PET, particularly in the early stage.^84,85^ The results of our FDG-PET analysis show that the D+E− group had significant hypermetabolism in the paracentral lobules and superior parietal gyrus, similar to the CAPPAH sign, and the regional hypermetabolism declined in the D+E+ group. This finding supports the observation that tight high convexity subarachnoid spaces is the first change on DESH findings.^86^ We propose that the CAPPAH sign reflects venous congestion over the high-convexity region, and the congested state decreases with the development of hydrocephalus. The significant hypometabolism in caudate was consistent with findings from another study.^33^ The mismatch between the hypometabolism in the striatum and hypermetabolism in the paracentral lobule was in agreement with prior studies, that more pronounced cerebral blood flow reduction adjacent to the lateral ventricles, and a logarithmic normalization occurring with distance from the ventricles.^87,88^ For the CAPPAH sign and caudate hypometabolism to co-occur, degeneration or insufficiency of deep venous system is considered as the rudimentary pathological mechanism. Chronic venous insufficiency is a condition of impaired venous blood flow causing venous hypertension with primary or secondary aetiologies, and aging is the most possible risk factor in the neurodegenerative diseases we addressed here.

An important supportive finding was the significantly increased PVH and DWM lesions in the D+E+ and D+E− groups, especially the Fazekas grade 3 DWM, a phenomenon that has long been observed in NPH patients.^28,89^ The histopathology of PVH and DWM hyperintensities is heterogeneous, mostly related to ischaemic-gliotic changes due to cerebral small vessel disease.^90^ It is associated with traditional vascular risk factors, subsequent stroke and cognitive impairment.^91-93^ Though hypertension is among the most common aetiologies causing small vessel damage, these lesions can be seen in normotensive patients as well.^94^ The venous blood of the supratentorial region is drained by two networks: the superficial (cortical) and the deep cerebral veins. Most superficial veins drain into the SSS, and most deep veins drain into the internal cerebral vein (ICV).^95^ We noted during analysis that the distribution of DWM lesions seems to be within the ICV territory and the lesions with a Fazekas score of 3 are in the watershed of cortical and deep venous systems (an example of axial imaging from one D+E+ participant is shown in Supplementary Fig. 4A, and the cartoon imaging of cerebral venous territory in Supplementary Fig. 4B is a clip).^80,96^ Although these observations would need to be validated in a comprehensive analysis, they strengthened our proposal that deep venous insufficiency is the cause of DESH.

Unlike systemic veins, the cerebral veins and sinuses have no tunica muscularis or valves, and could be readily collapsed under small changes in transmural pressure.^97^ They have bidirectional flow and thus are largely anastomosed to each other. The impaired deep venous system causes congestion of ICV, gradually spreading to the superficial venous system through transmedullary veins,^98^ leading to decreased venous return to SSS, and the collapsed SSS disturbs CSF absorption. This vicious cycle is presumed to be the aetiology of hydrocephalus development. These speculations were supported by one study that showed reduced cortical vein compliance, decreased drainage from the SSS, and reduced CSF resorption into the SSS, in NPH.^99^ In our FDG-PET results, the sequential change of hypermetabolic state in the paracentral lobules and superior parietal gyrus in D+E− and D+E+ groups reaffirms the later development of hydrocephalus than the tight high convexity subarachnoid spaces.

Another important finding in this study was the enlarged crural, ambient and quadrigeminal cisterns in D+E+ participants. An imaging proof of direct CSF communication between lateral ventricles and cisterns in subarachnoid space was provided in Supplementary Fig. 5. These results are consistent with one study that used high-field 3D MRI and showed that the choroidal fissure is responsible for the disproportionate CSF distribution in NPH.^25,26^ The D+E+ group comprises mostly PSP participants, and midbrain atrophy is a characteristic feature of PSP. Indeed, greater midbrain atrophy was noted in D+E+ participants. We hypothesize that the atrophic midbrain expands cisterns around the choroidal fissure, and this contour change leads to opening of the choroidal fissure. Through the choroidal fissure opening, CSF may overflow from the ventricular system to the subarachnoid space. Either from choroid plexus in the classic theory or cerebral capillaries in the modern theory, CSF is continuously secreted, or the continuous CSF production keeps expanding the crural/ambient/quadrigeminal cisterns. These cisterns are close to the Sylvian fissures and connect mutually through subarachnoid space; the outflow of CSF from the enlarged cisterns further dilates the Sylvian fissures. According to the Monro–Kellie doctrine, change in the brain parenchyma, blood or CSF volume will result in the reciprocal change in others to maintain the constancy within the cranium.^100^ Both in the classic theory that CSF is absorbed mainly through arachnoid granulations, and the alternative pathway through lymphatic system, the SSS has the most abundant arachnoid granulations and meningeal lymphatic vessels. CSF flow direction should be rostral-caudal to its absorption place around the SSS,^101^ and the crowded high vertex sulci is thus formed due to the Monro–Kellie doctrine along with the impaired CSF resorption of SSS that we mentioned in the prior paragraph. Superimposed on the theory of hydrocephalus caused by deep venous insufficiency mentioned previously, the role of midbrain atrophy and CSF outflow from choroidal fissure may enhance and promote the formation of DESH. This hypothesis will need more CSF dynamic testing to support and prove.

Diminishment of subarachnoid spaces in the high parietal convexity is a pivotal feature of DESH.^25^ Interestingly, there were no CBS participants in DESH positive groups. CBS patients usually present with asymmetric atrophy in the posterior frontal and parietal regions.^102^ Since the imaging hallmark of DESH presents symmetrically, it explains why CBS was absent from D+E+ and D+E− groups. Alzheimer’s disease frequently co-exists with NPH,^5,103^ and their characteristic picture is bilateral parietal atrophy. The disparity of neurological symptoms between Alzheimer’s disease and NPH and the definition of DESH (high tight parietal convexity), which negates the presence of bilateral parietal atrophy, could help in differentiating NPH from Alzheimer’s disease. Based on these observations and our assumptions, DESH is caused by deep cerebral venous insufficiency and is a transient phenomenon during the CSF outflow from the choroidal fissure, which could be a useful imaging feature to differentiate it from ‘neurodegenerative disease with parietal atrophy’, but should not be the sole determinant in making the decision to shunt given the low prevalence and its transient existence. Unlike ischaemia caused by arterial occlusion, venous insufficiency has a relatively indolent course and could be reversed with early intervention.^29^ The application of DESH on shunt decision results in inconsistent benefits.^104,105^ Prior long-term follow-up studies of NPH patients after shunting also revealed that several of them showed delayed deterioration of symptoms despite an initial improvement for the first months.^106,107^ For the high co-occurrence rate of PSP and NPH and the deep venous system degeneration as the main pathological mechanism, we suggest that when discussing shunt placement with hydrocephalic patients, more considerations, in addition to the response of CSF tap test, should be taken into account. Explaining to patients and families about the likelihood of overlapping Parkinsonism and the possibility of diminishing effects after a period is crucial.

The GMV measurement revealed smaller volumes of the medial orbitofrontal cortex and temporal poles in both the D+E+ and D−E+ groups, and broader regions were affected in the D−E+ group. These findings coincide with our theory that DESH develops earlier than hydrocephalus. Tight high convexity subarachnoid spaces are an early response to intra-cranial volume re-distribution, but significant GMV loss appears during the development of hydrocephalus. Whether the hydrocephalus is caused by grey matter atrophy or vice versa remains to be clarified. The reason for the larger volume of the superior parietal lobule in D+E+ participants is unclear but may partly be attributed to the lack of CBS in this group or segmentation inaccuracies driven by the deformed ventricles. The DTI analysis showed that the inferior frontal-occipital fasciculus, inferior longitudinal fasciculus, superior longitudinal fasciculus and uncinate fasciculus were more impaired in the D+E+ group. The inferior frontal-occipital fasciculus, inferior longitudinal fasciculus, and superior longitudinal fasciculus are long fibres passing through periventricular regions (Supplementary Fig. 6), thus impairment of these tracts in D+E+ participants is not surprising. These tracts were frequently reported to reflect the impaired structures in NPH or as predictors for shunt response.^59,60^ The degeneration of inferior longitudinal fasciculus, superior longitudinal fasciculus and uncinate fasciculus has been mentioned in PSP.^39,108^ The results reiterated the overlapped imaging features between the two diseases.

Our study measured several commonly used NPH indices. The Japanese NPH guidelines suggest that DESH should be evaluated in EI > 0.3 hydrocephalic patients.^12^ However, D+E− participants do exist in our study, and good response to shunt was ever reported in DESH-like but EI < 0.3 patient.^109^ In fact, the D+E− group may reflect our theory’s early stage of deep venous insufficiency. Though being used as a good tool to differentiate NPH from Alzheimer’s disease and a part of DESH characteristics,^110^ the traditional definition of CA < 90° in NPH seems too strict, and only eight cases met the criteria in our cohort of 238 participants (Supplementary Table 3). In addition, even the smallest CA in our DESH/EI groups, the median of the D+E+ group was 101.9°. EI is the most clinically accessible parameter for hydrocephalus measurement, though disease severity was worse, imaging features and GMVs of the D−E+ group were similar to those of the D+E+ group (Tables 1, 3, and 4). In our theory, hydrocephalus develops in the later stage of NPH, but the rarity of DESH, its transient existence, and the varied shunt response in DESH positive patients in prior reports,^111,112^ do not prevent clinicians using EI as a parameter to determine hydrocephalus and eligibility for shunt insertion.

The main limitation of this study was the cross-sectional design. To clarify the sequential development of imaging features and prove our theory on DESH, a longitudinal prospective study is mandatory. The lack of venous system-focused imaging is also a limitation, especially one capable of directly depicting the deep venous system. The relatively small sample size of the D+E− participants and of the participants with FDG-PET and with DTI, undermine the power of our analyses. The intra- and inter-rater reliability of PVH and DWM were relatively low, thus the interpretation of these analysis should be cautious. The fact that we lacked information about vascular risk factors limits our interpretations of the DWM findings. We used 0.3 as the cut-point of EI, but lower cut-off values for hydrocephalus have been proposed.^113^ The determination of DESH was positive if the score was >1.0, and whether excluding borderline cases causes selection bias remains questionable. Our grouping relied mainly on the clinical diagnosis, and lacking autopsy confirmation may obscure the definite diagnosis of reported cases.

The strengths of this study were the large number of PSP subjects, the application of automated and unbiased detection of DESH, and the detailed comparison between different DESH/EI groups to get a clearer picture of hydrocephalus. In our visually assessed DESH, the positive rate was 3.8%, and the automated DESH determined 11.8% positive. It is evident that machine-based method could catch minimal change of sulcus and fissure involved in high convexity, giving higher sensitivity of detection, however, this tool is not clinically accessible and its clinical application needs to be validated.

Our study reaffirmed the overlapped features in PSP and NPH, disclosed the characteristics of DESH, and proposed a mechanism for its formation. The degeneration of the deep cerebral venous system is considered the main pathophysiology of DESH, implying that aside from the traditionally emphasized arterial system, the venous system is also a sine qua non of brain health. We avoid using ‘idiopathic’ NPH here since accumulating evidence supports many neurodegenerative processes underlying this disease, and more studies are needed to provide the whole picture of NPH.

## Supplementary Material

fcaf206_Supplementary_Data

## Data Availability

All data from the study will be shared in an anonymized format to any qualified investigator on request from the corresponding author.
