# Correction to: Evaluation of disproportionately enlarged subarachnoid-space hydrocephalus in progressive supranuclear palsy

**DOI:** 10.1093/braincomms/fcaf465

**Published:** 2025-12-09

This is a **correction** to:

Mu-Hui Fu, Jeffrey L Gunter, Ryota Satoh, Rodolfo G Gatto, Farwa Ali, Heather M Clark, Julie A Stierwalt, Mary M Machulda, Yehkyoung C Stephens, Hossam Youssef, Nha Trang Thu Pham, Clifford R Jack, Val J Lowe, Keith A Josephs, Jennifer L Whitwell, Evaluation of disproportionately enlarged subarachnoid-space hydrocephalus in progressive supranuclear palsy, *Brain Communications*, Volume 7, Issue 3, 2025, fcaf206, https://doi.org/10.1093/braincomms/fcaf206

In the originally published version of this manuscript, there was an error in the Clinical demographics of DESH/EI groups Section. The original sentence reads as follows: “Of the 18 PSP participants, the most common clinical variant was PSP-RS, occurring in 50% of the group…”. This percentage is incorrect – it should be 45%.

The correct sentence is as follows:

“Of the 18 PSP participants, the most common clinical variant was PSP-RS, occurring in **45%** of the group”.

This does not impact any conclusions in this paper, and the error has been corrected.

