# Correction to: Evaluation of disproportionately enlarged subarachnoid-space hydrocephalus in progressive supranuclear palsy

**DOI:** 10.1093/braincomms/fcag297

**Published:** 2026-08-03

This is a correction to: Mu-Hui Fu, Jeffrey L Gunter, Ryota Satoh, Rodolfo G Gatto, Farwa Ali, Heather M Clark, Julie A Stierwalt, Mary M Machulda, Yehkyoung C Stephens, Hossam Youssef, Nha Trang Thu Pham, Clifford R Jack, Val J Lowe, Keith A Josephs, Jennifer L Whitwell, Evaluation of disproportionately enlarged subarachnoid-space hydrocephalus in progressive supranuclear palsy, *Brain Communications*, Volume 7, Issue 3, 2025, fcaf206, https://doi.org/10.1093/braincomms/fcaf206

In the originally published version of this manuscript, the last group of Figure 2 contained an error.

This has been corrected to read “DESH(-)EI(-) n=115” instead of “DESH(-)EI(+) n=115”.

