# Supplementary material for: Evaluation of disproportionately enlarged subarachnoid-space hydrocephalus in progressive supranuclear palsy
Source: Brain Commun. 2025 Jun 3;7(3):fcaf206. doi: 10.1093/braincomms/fcaf206 (PMC12152481; doi:10.1093/braincomms/fcaf206)
Supplement: fcaf206_Supplementary_Data [file fcaf206_supplementary_data.docx]

**Supplementary Table 1: MRI scanner acquisition parameters**

|  | **GE scanner** | **Siemens scanner** |
| --- | --- | --- |
| **Field Strength** | 3 Tesla | 3 Tesla |
| **MPRAGE** |  |  |
| Acceleration | None | Yes |
| Orientation | Sagittal | Sagittal |
| Voxel size | 1.2 x 1 x 1mm | 0.8 x 0.8 x 0.8mm |
| Field of view (FOV) | 199 x 260 x 260 mm | 192 x 240 x 256mm |
| Flip angle | 8° | 9° |
| Repetition time (TR) | 2300ms | 2300ms |
| Echo time (TE) | 3ms | 3.14ms |
| T1 relaxation time | 900ms | 945ms |
| **FLAIR** |  |  |
| Orientation | Axial | Sagittal |
| Voxel size | 0.86 x 0.86 x 3.6mm | 1.2 x 1 x 1mm |
| Field of view (FOV) | 220 x 220 x 147 mm | 192 x 256 x 256mm |
| Flip angle | 90 | 120 |
| Repetition time (TR) | 11000ms | 4800ms |
| Echo time (TE) | 11000ms | 441ms |
| Inversion Time (TI) | 2250ms | 1500ms |
| **Diffusion MRI** |  |  |
| Orientation | Axial | NA |
| Voxel size | 1.37 x 1.37 x 2.7mm | NA |
| In plane resolution | 2.7mm | NA |
| Field of view (FOV) | 231 x 350 x 159mm | NA |
| Flip angle | 90° | NA |
| Repetition time (TR) | 11,300 or 8000ms | NA |
| Echo time (TE) | 67.8 or 57.3ms | NA |
| Phase Acceleration | 2 | NA |
| Diffusion directions | 41 | NA |
| b values | 5 b=0  41 b=1000 s/mm^2^ | NA |

NA = not applicable since all DTI analyses were performed using GE scans

**Supplementary Table 2. Forty-seven regions of gray matter volumes analysis**

| **ROI (total)** | |
| --- | --- |
| Amygdala | Inferior Occipital Gyrus |
| Angular Gyrus | Middle Occipital Gyrus |
| Calcarine Cortex | Superior Occipital Gyrus |
| Caudate Nucleus | Olfactory Cortex |
| Anterior Cingulate Gyrus | Pallidum |
| Middle Cingulate Gyrus | Paracentral Lobule |
| Posterior Cingulate Gyrus | Parahippocampal Gyrus |
| Cuneus | Inferior Parietal Lobule |
| Entorhinal Cortex | Superior Parietal Lobule |
| Inferior Frontal Operculum | Postcentral Gyrus |
| Inferior Frontal Orbital Cortex | Precentral Gyrus |
| Inferior Frontal Triangularis | Precuneus |
| Medial Orbital Frontal Cortex | Putamen |
| Middle Frontal Gyrus | Rectus Gyrus |
| Middle Orbital Frontal Cortex | Retrosplenial Cortex |
| Superior Frontal Gyrus | Rolandic Operculum |
| Superior Medial Frontal Cortex | Supplementary Motor Area |
| Superior Orbital Frontal Cortex | Supramarginal Gyrus |
| Fusiform Gyrus | Inferior Temporal Gyrus |
| Heschl's Gyrus | Middle Temporal Gyrus |
| Hippocampus | Temporal Pole of the Middle Temporal Gyrus |
| Insula | Temporal Pole of the Superior Temporal Gyrus |
| Lingual Gyrus | Superior Temporal Gyrus |
|  | Thalamus |

**Supplementary Table 3. Demographic data of different Parkinsonism groups and controls**

| Variable | PSP  (N=181) | CBS  (N=36) | PD  (N=21) | Control  (N=52) | FDR corrected p values | Only PSP, CBS, PD |
| --- | --- | --- | --- | --- | --- | --- |
| Female, n (%) | 83 (46%) | 17 (47%) | 6 (28.5%) | 33 (59%) | 0.13 | 0.28 |
| Age at encounter, yr | 70.3 (65.8, 75.5) | 67.7 (62.8, 71.9) | 69.1 (65.7, 77.8) | 68.4 (65.8, 71.5) | 0.85 | 0.69 |
| MoCA (30) | 23 (19, 26) | 22 (20, 24) | 25 (23, 27) | 27 (26, 28) | **<0.001**^c, e, f^ | 0.06 |
| UPDRS-III (132) | 39 (30, 52) | 30 (23, 41) | 25.5 (18, 44) | 0 (0, 0) | **<0.001**^c, e, f^ | **0.004**^a^ |
| PSP Rating Scale (100) | 37 (28, 45) | 27 (22, 40) | 12 (7, 18) | 0 (0, 0) | **<0.001**^b, c, d, e^ | **<0.001**^b, d^ |
| PSIS (5) | 3 (2, 3) | 1 (0, 1) | 0 (0, 1) | 0 (0, 0) | **<0.001**^a, b, c, e^ | **<0.0001**^a, b^ |
| Tulia (12) | 11 (9, 12) | 5 (2, 10) | 12 (12, 12) | 12 (12, 12) | **<0.001**^a, b, c, d, e^ | **<0.0001**^a, b, d^ |
| EI > 0.3 | 83 (45.8%) | 13 (36.1%) | 7 (33.3%) | 7 (13.5%) | **0.006**^c, e^ | 0.35 |
| CA < 90° | 7 (3.9%) | 1 (2.8%) | 0 | 0 | 0.46 | 0.63 |
| Automated DESH positive | 25 (13.8%) | 0 (0%) | 3 (14.3%) | 3 (5.8%) |  |  |
| Visual DESH positive | 8 (4.4%) | 0 (0%) | 1 (4.8%) | 0 (0%) |  |  |

Data shown as n (%) or median (range). Categorical variables were compared across groups using chi-square tests (Fishers exact test for cells with small numbers) and continuous variables were compared using Kruskal-Wallis tests. False Discovery Rate (FDR) correction was applied to control for multiple comparisons. Pair-wise comparisons were corrected for multiple comparisons using Dunn’s test. All significant differences are highlighted in bold. CA, callosal angle; EI, Evans’ index; MoCA, Montreal cognitive assessment; PSIS, PSP saccadic impairment scale; Tulia, apraxia screen of Tulia; UPDRS-III, Unified Parkinson’s Disease Rating Scale Part III. Superscript alphabet(s) in *P*-value represents: ^a^ PSP versus CBS; ^b^ PSP versus PD; ^c^ PSP versus Control; ^d^ CBS versus PD; ^e^ CBS versus Control; ^f^ PD versus Control. Bold values indicate statistical significance (P < 0.05).

**Supplementary Table 4. Distribution of two MRI scanners**

|  | DESH(+)EI(+)  (N=20) | DESH(+)EI(-)  (N=8) | DESH(-)EI(+)  (N=71) | DESH(-)EI(-)  (N=114) | *P* |
| --- | --- | --- | --- | --- | --- |
| **GE** | 12 (60%) | 3 (37%) | 31 (44%) | 36 (32%) | 0.07 |
| **Siemens** | 8 (40%) | 5 (63%) | 40 (56%) | 78 (68%) |  |

Data shown as n; Chi-square test was used for comparison.

**Supplementary Table 5. Demographic data of the four DESH/EI (D/E) groups (only PSP participants)**

| Variable | D+E+  (N=18) | D+E-  (N=7) | D-E+  (N=55) | D-E-  (N=82) | FDR corrected p values |
| --- | --- | --- | --- | --- | --- |
| Female, n (%) | 7 (38.9%) | 5 (71.4%) | 16 (29%) | 46 (56%) | **0.03**^d,f^ |
| Age at encounter, yr | 71.9 (67.6, 75.7) | 69.5 (62.8, 73.6) | 70.8 (67.8, 76.2) | 69.2 (64.8, 73.7) | 0.28 |
| Age at onset, yr | 67.5 (63.5, 71) | 65 (54, 70) | 67 (63.5, 72) | 64.9 (59, 70) | 0.31 |
| Disease duration, yr | 3.65 (2.35, 7.9) | 5 (3.45, 7.8) | 3.6 (2.35, 4.6) | 3.3 (2.4, 5.2) | 0.61 |
| MoCA (30) | 23 (19.8, 25.8) | 23 (18, 26) | 22 (19, 24.8) | 24 (22, 26) | 0.25 |
| UPDRS-III (132) | 42.5 (30.3, 53.8) | 38 (34, 51.5) | 42 (32.5, 57.5) | 36 (26.5, 46.8) | 0.24 |
| PSIS (5) | 2.5 (1, 3) | 3 (3, 4) | 3 (2, 3.5) | 3 (1, 3) | 0.26 |
| FAB (18) | 13 (12, 14.8) | 16 (13, 17) | 13.5 (11, 15) | 14 (12, 16) | 0.29 |
| ASRS (52) | 3.5 (1, 6.25) | 4 (2.75, 6.25) | 4 (2, 8) | 4 (2, 6) | 0.79 |
| PSP rating scale (100) | 36 (27.8, 41.5) | 38 (33.5, 49.5) | 38 (31, 49.5) | 34 (27, 43.8) | 0.21 |
| PSP Rating Scale_GM (20) | 14 (9.3, 15) | 10 (8.5, 14) | 12 (7, 14.5) | 11 (5, 14) | 0.58 |
| *PSP Rating Scale downgaze score* | 1.5 (1, 3.75) | 3.5 (3, 4) | 3 (2, 4) | 3 (1, 4) | **0.03**^a,b,c^ |
| PSP Rating Scale downgaze positive (score ≥ 1), % | 100 | 100 | 96.3 | 84 |  |
| *PSP Rating Scale disorientation score* | 0.5 (0, 1) | 0 (0, 0) | 0 (0, 1) | 0 (0, 0) | **0.035**^c^ |
| PSP Rating Scale disorientation positive (score ≥ 1), % | 50 | 16.7 | 35.2 | 16 |  |
| *PSP Rating Scale urinary incontinence score* | 0.5 (0, 3) | 3 (2.25, 3.75) | 1 (0, 3) | 0 (0, 3) | 0.69 |
| PSP Rating Scale urine incontinence positive (score ≥ 1), % | 50 | 83.3 | 55.6 | 45.7 |  |

Data shown as n (%) or median (range); Categorical variables were compared across groups using chi-square tests (Fishers exact test for cells with small numbers) and continuous variables were compared using Kruskal-Wallis tests. False Discovery Rate (FDR) correction was applied to control for multiple comparisons. Pair-wise comparisons were corrected for multiple comparisons using Dunn’s test. ASRS, apraxia of speech rating scale; FAB, frontal assessment battery; MoCA, Montreal cognitive assessment; PSIS, PSP saccadic impairment scale; PSP Rating Scale_GM, PSP Rating Scale gait/midline subscore; UPDRS-III, Unified Parkinson’s Disease Rating Scale Part III. Superscript alphabet(s) in *P*-value represents:^a^ DESH(+)EI(+) versus DESH(+)EI(-); ^b^ DESH(+)EI(+) versus DESH(-)EI(+); ^c^ DESH(+)EI(+) versus DESH(-)EI(-); ^d^ DESH(+)EI(-) versus DESH(-)EI(+); ^e^ DESH(+)EI(-) versus DESH(-)EI(-); ^f^ DESH(-)EI(+) versus DESH(-)EI(-). Bold values indicate statistical significance (P < 0.05).

**Supplementary Table 6. Imaging features of the four DESH/EI (D/E) groups (only PSP participants)**

| Parameter | D+E+  (N=18) | D+E-  (N=7) | D-E+  (N=55) | D-E-  (N=82) | FDR corrected p values |
| --- | --- | --- | --- | --- | --- |
| CA | 97.7 (89.9, 114.2) | 121.3 (103.2, 124.6) | 115.4 (109.2, 123.2) | 121.3 (113.7, 125.6) | **<0.001^a,b,c,f^** |
| MRPI | 21.15 (17.26, 23.89) | 24.50 (17.77, 29.23) | 20.15 (14.47, 25.42) | 14.89 (11.49, 18.32) | **<0.001^c,e,f^** |
| ALVI | 0.47 (0.45, 0.55) | 0.45 (0.41, 0.46) | 0.46 (0.43, 0.50) | 0.44 (0.41, 0.47) | **<0.001^a,b,c,f^** |
| Cistern areas (cm^2^) | 7.77 (7.20, 8.55) | N/A | N/A | 6.06 (5.66, 6.55) | **<0.001^c^** |
| Fazekas score |  |  |  |  |  |
| PVH 1 | 4 (22.2%) | 2 (28.6%) | 13 (23.6%) | 43 (52.4%) | **0.02^c,f^** |
| PVH 2 | 9 (50%) | 3 (42.8%) | 31 (56.4%) | 29 (35.4%) |  |
| PVH 3 | 5 (27.8%) | 2 (28.6%) | 11 (20%) | 10 (12.2%) |  |
| Average PVH score | 2.06 | 2.00 | 1.96 | 1.60 |  |
| DWM 0 | 1 (5.5%) | 1 (14.3%) | 10 (18.2%) | 21 (25.6%) | 0.06 |
| DWM 1 | 9 (50%) | 3 (42.9%) | 28 (50.9%) | 43 (52.4%) |  |
| DWM 2 | 5 (27.8%) | 2 (28.5%) | 17 (30.9%) | 15 (18.3%) |  |
| DWM 3 | 3 (16.7%) | 1 (14.3%) | 0 (0%) | 3 (3.7%) |  |
| Average DWM score | 1.56 | 1.43 | 1.13 | 1.00 |  |

Data shown as n (%) or median (range). Group comparisons of CA, MRPI, and ALVI were performed using linear regression adjusting for age and gender, and False Discovery Rate (FDR) correction was applied to control for multiple comparisons. Categorical variables were compared across groups using chi-square tests (Fishers exact test for cells with small numbers) with FDR correction for multiple comparisons. ALVI, anteroposterior diameter of the lateral ventricle index; CA, callosal angle; DWM, deep white matter hyperintensities; MRPI, MR parkinsonism index; PVH, periventricular hyperintensities. Cistern includes crural, ambient, and quadrigeminal cisterns. N/A is not assessed. Superscript alphabet(s) in *P*-value represents:^a^ DESH(+)EI(+) versus DESH(+)EI(-); ^b^ DESH(+)EI(+) versus DESH(-)EI(+); ^c^ DESH(+)EI(+) versus DESH(-)EI(-); ^d^ DESH(+)EI(-) versus DESH(-)EI(+); ^e^ DESH(+)EI(-) versus DESH(-)EI(-); ^f^ DESH(-)EI(+) versus DESH(-)EI(-). Bold values indicate statistical significance (P < 0.05).

**Supplementary Table 7. MRI region-of-interest volumes of the four DESH/EI (D/E) groups (only PSP participants)**

| ROI | D+E+  (N=18) | D+E-  (N=7) | D-E+  (N=55) | D-E-  (N=82) | P value adjusted for age and gender |
| --- | --- | --- | --- | --- | --- |
| **Frontal lobe** |  |  |  |  |  |
| Medial orbitofrontal cortex | 0.37 (0.32, 0.41) | 0.37 (0.35, 0.38) | 0.38 (0.35, 0.42) | 0.42 (0.37, 0.46) | **<0.001** |
| Middle frontal gyrus orbital part | 0.44 (0.39, 0.47) | 0.44 (0.39, 0.47) | 0.44 (0.42, 0.49) | 0.47 (0.42, 0.49) | **0.008** |
| Inferior frontal gyrus, triangular part | 0.47 (0.43, 0.53) | 0.48 (0.44, 0.54) | 0.49 (0.42, 0.53) | 0.51 (0.46, 0.57) | **0.002** |
| Inferior frontal gyrus, opercular part | 0.38 (0.34, 0.44) | 0.41 (0.40, 0.44) | 0.39 (0.34, 0.44) | 0.43 (0.38, 0.47) | **0.005** |
| **Temporal lobe** |  |  |  |  |  |
| Temporal pole, middle | 0.39 (0.38, 0.43) | 0.42 (0.36, 0.44) | 0.42 (0.39, 0.47) | 0.46 (0.41, 0.52) | **0.001^c,f^** |
| Superior temporal gyrus | 1.16 (1.08, 1.26) | 1.26 (1.11, 1.36) | 1.24 (1.10, 1.31) | 1.27 (1.18, 1.39) | **0.004** |
| **Parietal lobe** |  |  |  |  |  |
| Superior parietal gyrus | 0.83 (0.72, 0.90) | 0.71 (0.69, 0.77) | 0.74 (0.69, 0.81) | 0.80 (0.71, 0.89) | **0.002** |
| Inferior parietal gyrus | 0.41 (0.36, 0.51) | 0.43 (0.39, 0.49) | 0.39 (0.35, 0.43) | 0.43 (0.38, 0.47) | **0.001** |
| **Subcortical** |  |  |  |  |  |
| Thalamus | 0.33 (0.30, 0.36) | 0.38 (0.33, 0.39) | 0.34 (0.31, 0.36) | 0.37 (0.34, 0.39) | **0.003^f^** |
| **Insula** |  |  |  |  |  |
| Insula | 0.84 (0.79, 0.91) | 0.87 (0.84, 0.97) | 0.85 (0.80, 0.90) | 0.88 (0.84, 0.94) | **<0.001** |

Data shown as median (range); Group comparisons were performed using linear regression adjusting for age and gender, and False Discovery Rate (FDR) correction was applied to control for multiple comparisons. ROI volumes are expressed as percentage of TIV. Superscript alphabet(s) in *P*-value represents:^a^ DESH(+)EI(+) versus DESH(+)EI(-); ^b^ DESH(+)EI(+) versus DESH(-)EI(+); ^c^ DESH(+)EI(+) versus DESH(-)EI(-); ^d^ DESH(+)EI(-) versus DESH(-)EI(+); ^e^ DESH(+)EI(-) versus DESH(-)EI(-); ^f^ DESH(-)EI(+) versus DESH(-)EI(-). Bold values indicate statistical significance (P < 0.05).

**Supplementary Table 8. FDG SUVR of the four DESH/EI (D/E) groups (only PSP participants)**

| ROI | D+E+  (N=10) | D+E-  (N=5) | D-E+  (N=34) | D-E-  (N=54) | P value adjusted for age and gender |
| --- | --- | --- | --- | --- | --- |
| Paracentral lobule | 3.17 (3.02, 3.40) | 3.44 (3.30, 3.55) | 3.04 (2.90, 3.28) | 3.07 (2.93, 3.26) | **0.01^d,e^** |
| Superior parietal gyrus | 3.25 (3.19, 3.34) | 3.56 (3.46, 3.80) | 3.18 (3.04, 3.32) | 3.34 (3.13, 3.56) | **0.02^d^** |
| Precuneus | 3.31 (3.19, 3.43) | 3.68 (3.54, 3.77) | 3.33 (3.19, 3.52) | 3.51 (3.25, 3.75) | 0.26 |
| Caudate | 2.50 (2.41, 2.58) | 2.82 (2.81, 2.91) | 2.65 (2.50, 2.77) | 2.81 (2.73, 2.96) | **<0.001^a,b,c,f^** |
| Putamen | 2.77 (2.58, 2.87) | 3.11 (2.95, 3.21) | 2.86 (2.77, 2.99) | 2.94 (2.81, 3.07) | 0.10 |

Data shown as median (range). Group comparisons were performed using linear regression adjusting for age and gender, and False Discovery Rate (FDR) correction was applied to control for multiple comparisons. Superscript alphabet(s) in *P*-value represents:^a^ DESH(+)EI(+) versus DESH(+)EI(-); ^b^ DESH(+)EI(+) versus DESH(-)EI(+); ^c^ DESH(+)EI(+) versus DESH(-)EI(-); ^d^ DESH(+)EI(-) versus DESH(-)EI(+); ^e^ DESH(+)EI(-) versus DESH(-)EI(-); ^f^ DESH(-)EI(+) versus DESH(-)EI(-). Bold values indicate statistical significance (P < 0.05).


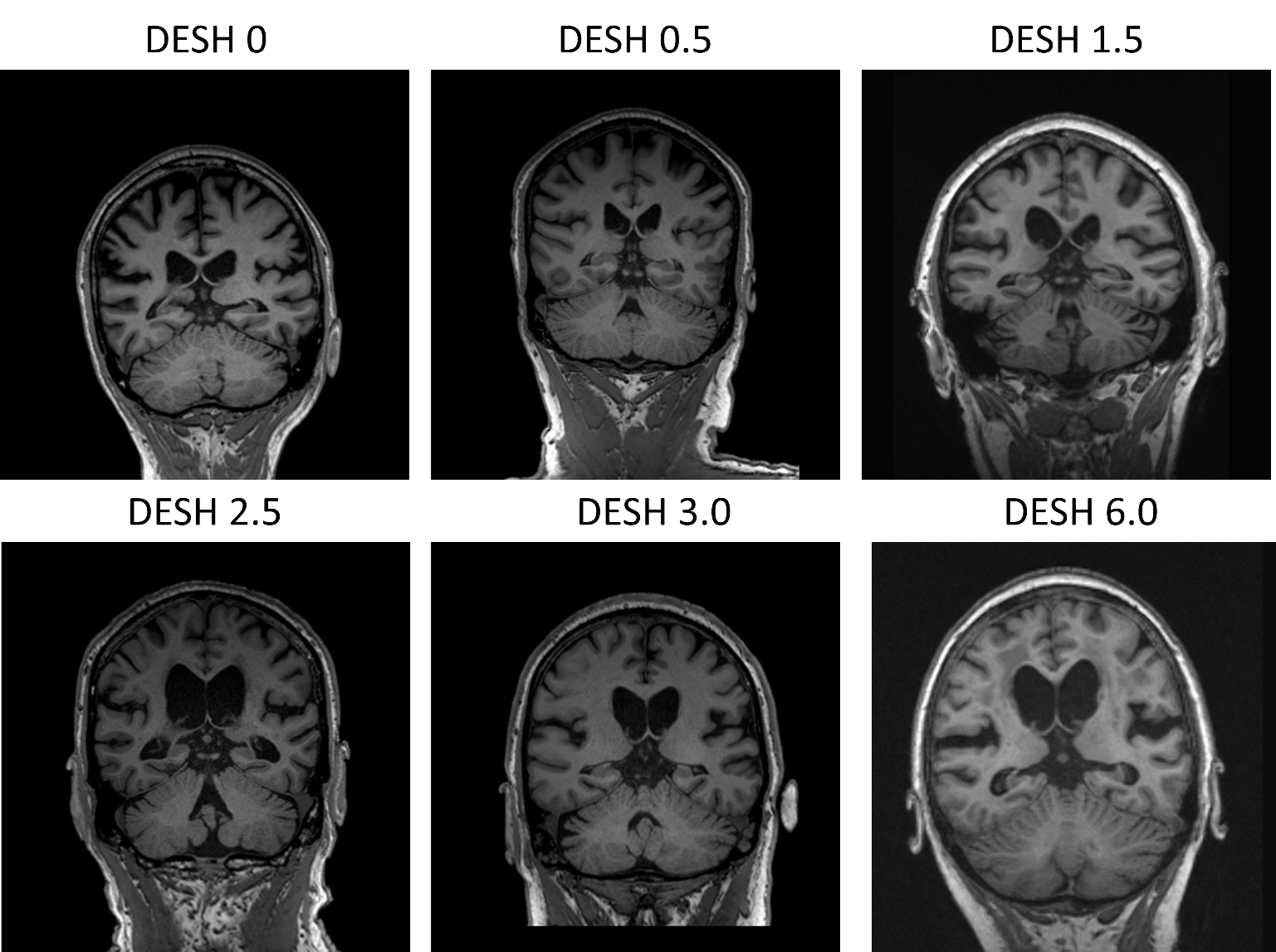


**Supplementary Figure 1. Example MRI coronal images of various automated DESH pattern scores.** Score > 1 were defined as positive DESH. DESH 6.0 was also visually assessed positive.

**
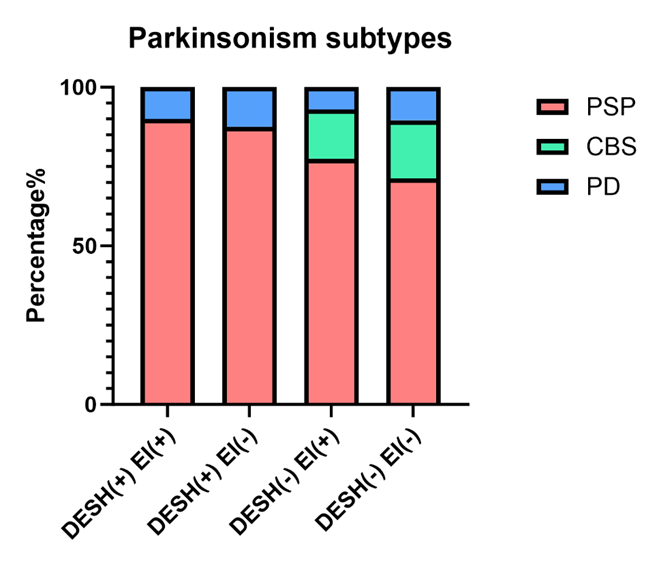
**

**Supplementary Figure 2. Parkinsonism subtypes of DESH/EI groups.** Bars show the percentage of cases in each DESH/EI defined group that were diagnosed with progressive supranuclear palsy (PSP) (red), corticobasal syndrome (CBS) (green) or Parkinson’s disease (PD) (blue).


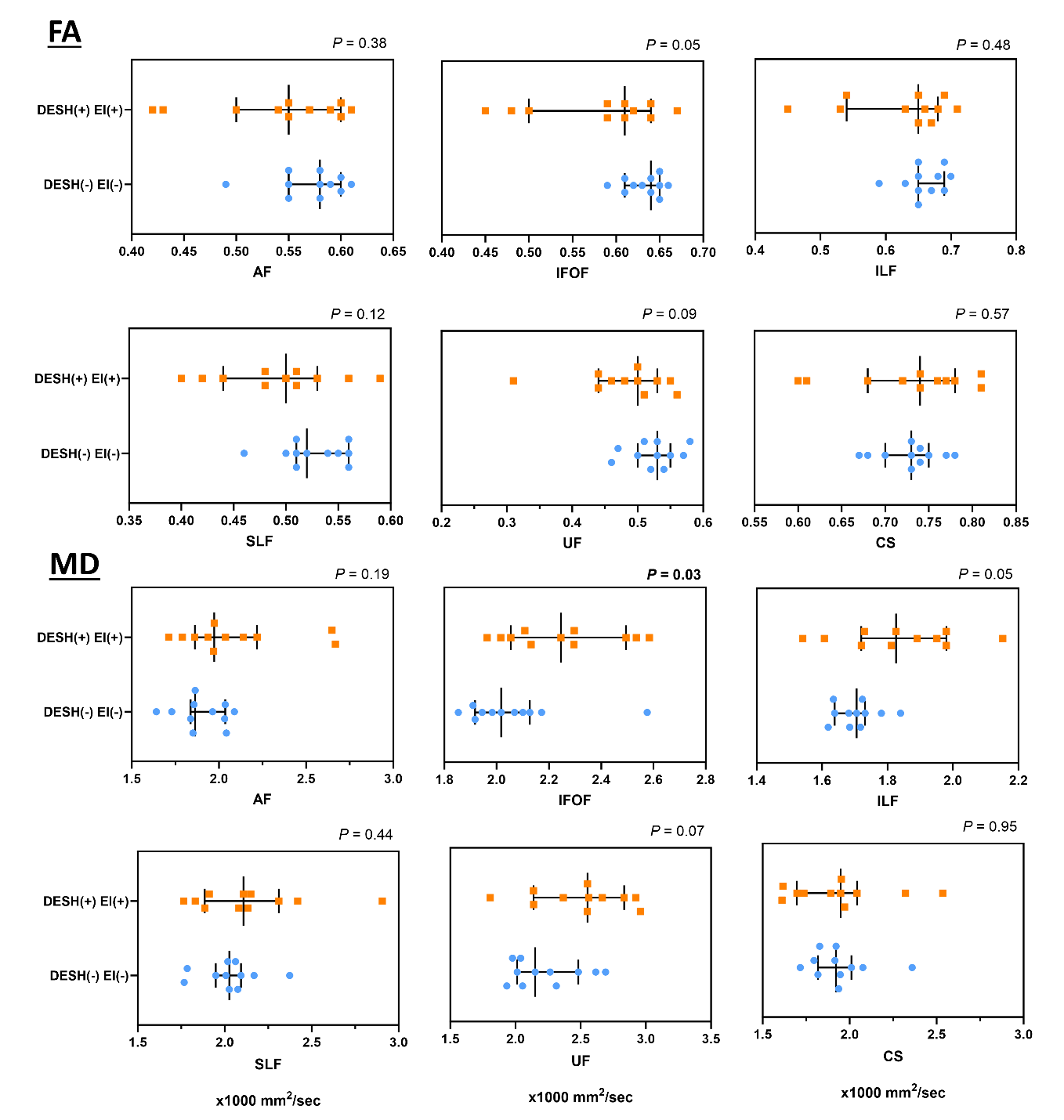


**Supplementary Figure 3. Results of white matter tracts in D+E+ and D-E- only PSP participants.** These plots show FA and MD metric for only PSP participants in the D+E+ and D-E- group, and present median with interquartile range. *P*-value is presented at right upper corner of each tract, and marked as bold if reaching significance. AF, arcuate fasciculus; CS, corticospinal tract; IFOF, inferior fronto-occipital fasciculus; ILF, inferior longitudinal fasciculus; UF, uncinate fasciculus. Each group contains eleven participants and were compared using Mann-Whitney U test.


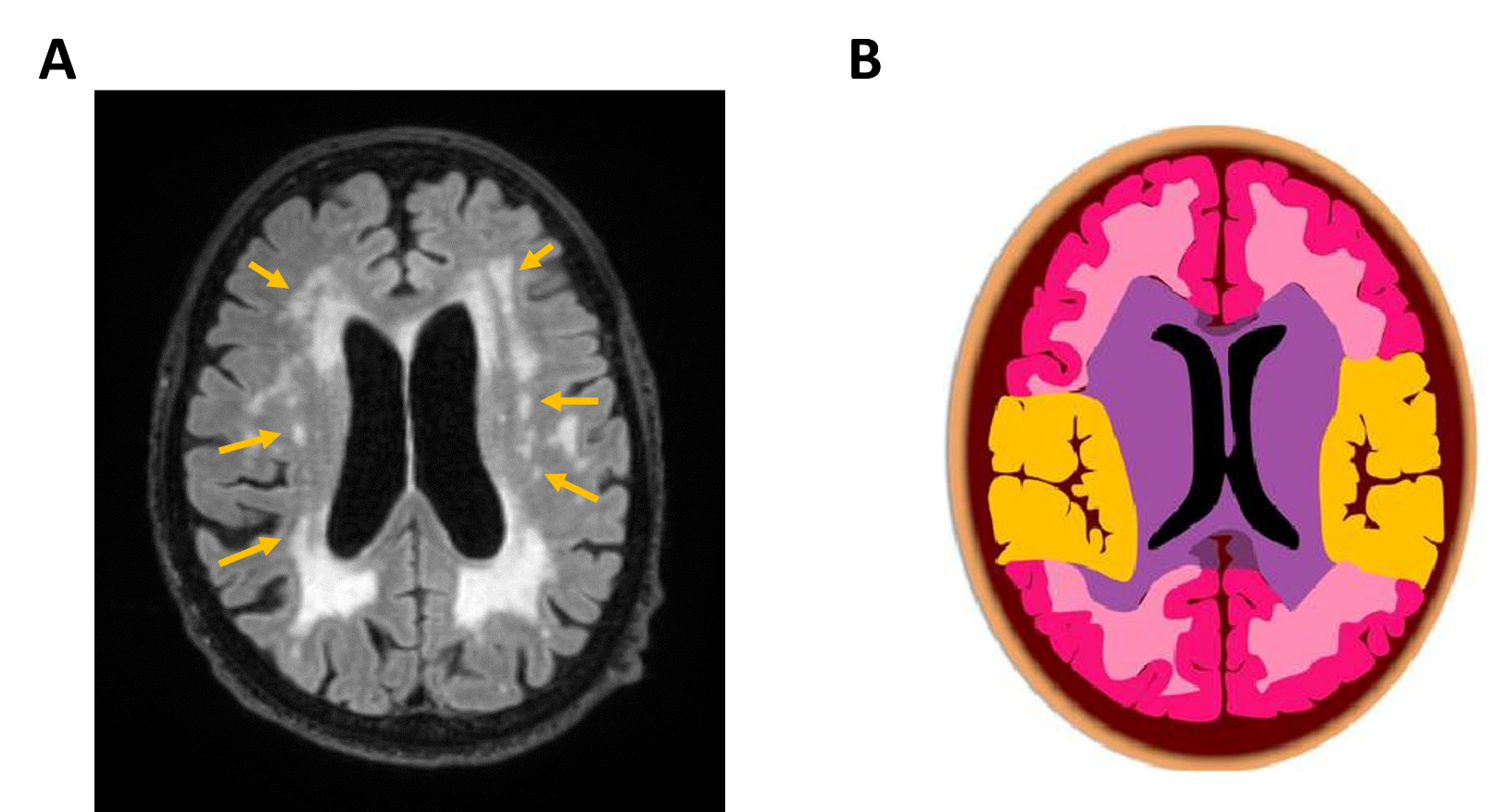


**Supplementary Figure 4. Distribution of deep white matter (DWM) lesions matches the location of the internal cerebral vein (ICV) territory.** (A) An axial FLAIR image from a participant in group D+E+. Orange arrows point to the DWM hyperintensities. (B) Schematic showing the location of different venous territories. The purple region marks the territory of the ICV, deep pink marks the territory of cortical veins, yellow marks the territory of the sphenoparietal sinus, and light pink denotes white matter.^1^


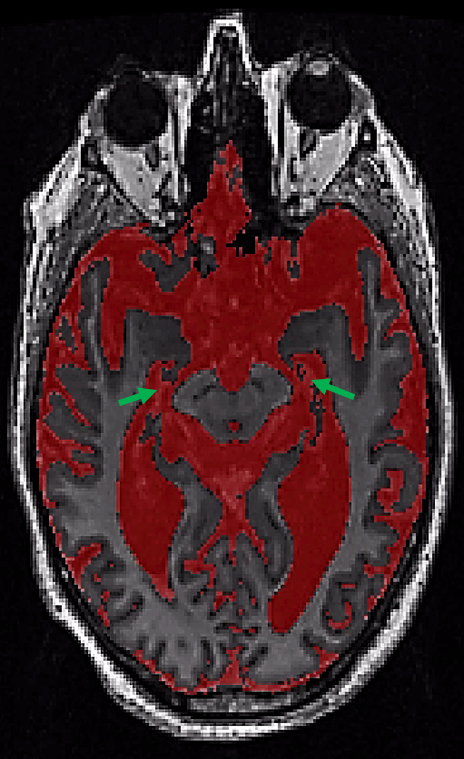


**Supplementary Figure 5. An axial T1-weighted MPRAGE image of a participant in the D+E+ group highlighting the choroidal fissure opening.** The cerebrospinal fluid segmentation is shown in red, and the green arrows highlight the opening of choroidal fissure which may cause direct cerebrospinal fluid communication between the lateral ventricles and cisterns in subarachnoid space.


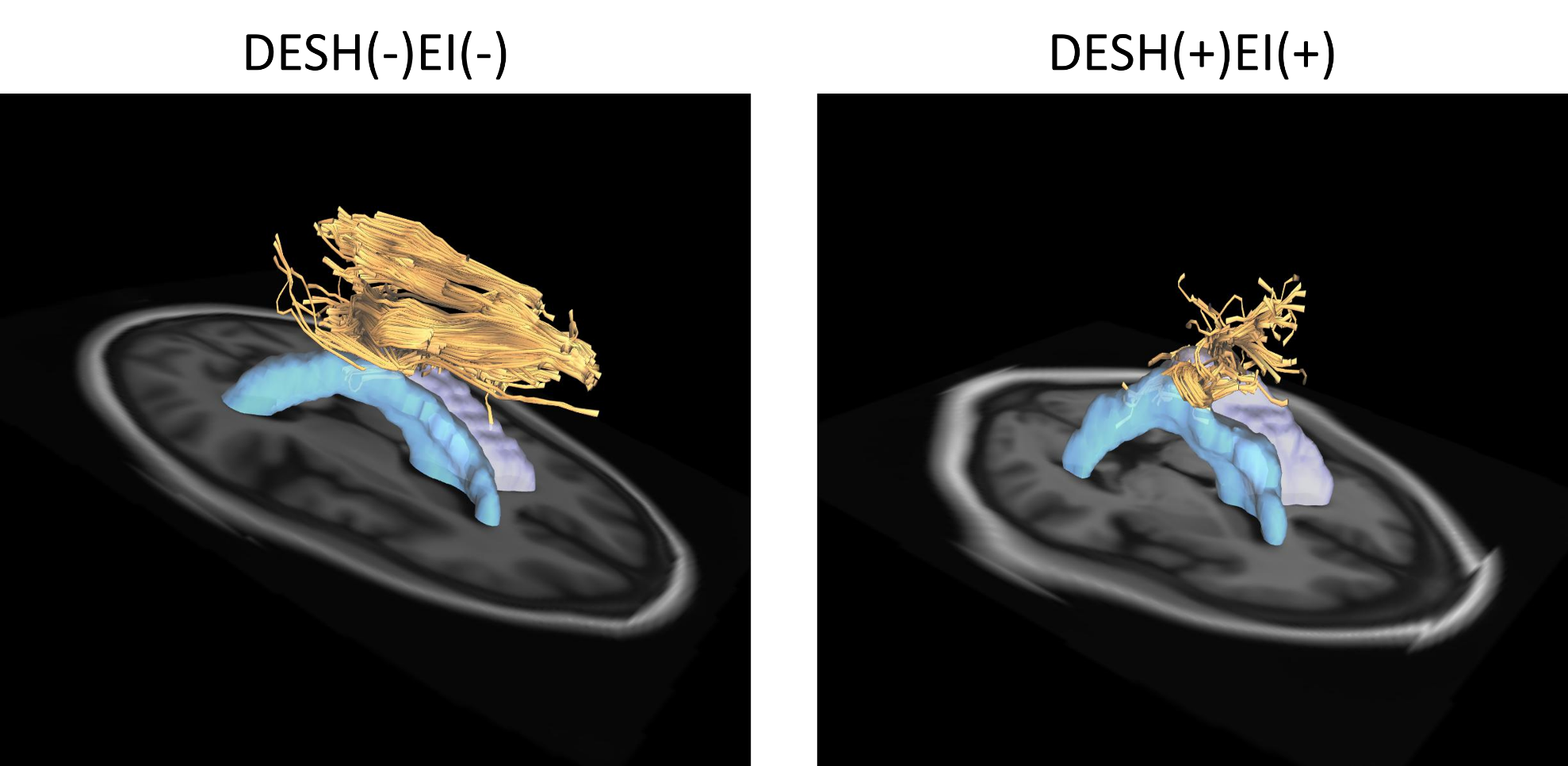


**Supplementary Figure 6. A three-dimensional rendering of the superior longitudinal fasciculus identified using tractography in a D-E- and D+E+ patient.** The rendering is shown in Mayo Clinic Adult Lifespan Template (MCALT) (<https://www.nitrc.org/projects/mcalt/>) space with a representative axial slice of the T1-weighted template shown. The blue and purple regions show a three-dimensional rendering of the lateral ventricles for reference, created using Freesurfer segmentations and rendered in DSI studio. DESH, disproportionately enlarged subarachnoid-space; EI, Evans’ index.

Reference:

1. García MC. What emergency and general radiologists should know about vascular (potentially) reversible brain disorders: Cerebral venus thrombosis (CVT), Posterior Reversible Encephalopathy Syndrome (PRES) and Reversible Cerebral vasoconstriction Syndrome (RCVS). 2015:
